# Role of Globotriaosylceramide in Physiology and Pathology

**DOI:** 10.3389/fmolb.2022.813637

**Published:** 2022-02-23

**Authors:** Ana Beatriz Celi, Jorge Goldstein, María Victoria Rosato-Siri, Alipio Pinto

**Affiliations:** ^1^ Laboratorio de Neurofisiopatología, Instituto de Fisiología y Biofísica “Houssay”, CONICET, Universidad de Buenos Aires, Buenos Aires, Argentina; ^2^ Departamento de Fisiología, Facultad de Medicina, Universidad de Buenos Aires, Buenos Aires, Argentina; ^3^ Departamento de Física Médica/Instituto de Nanociencia y Nanotecnología, Centro Atómico Bariloche, San Carlos de Bariloche, Argentina

**Keywords:** glycosphingolipids, Gb3, hemolytic uremic syndrome, cancer, glycosphingolipid physiology, glycosphingolipid pathology

## Abstract

At first glance, the biological function of globoside (Gb) clusters appears to be that of glycosphingolipid (GSL) receptors for bacterial toxins that mediate host-pathogen interaction. Indeed, certain bacterial toxin families have been evolutionarily arranged so that they can enter eukaryotic cells through GSL receptors. A closer look reveals this molecular arrangement allocated on a variety of eukaryotic cell membranes, with its role revolving around physiological regulation and pathological processes. What makes Gb such a ubiquitous functional arrangement? Perhaps its peculiarity is underpinned by the molecular structure itself, the nature of Gb-bound ligands, or the intracellular trafficking unleashed by those ligands. Moreover, Gb biological conspicuousness may not lie on intrinsic properties or on its enzymatic synthesis/degradation pathways. The present review traverses these biological aspects, focusing mainly on globotriaosylceramide (Gb3), a GSL molecule present in cell membranes of distinct cell types, and proposes a wrap-up discussion with a phylogenetic view and the physiological and pathological functional alternatives.

## 1 Introduction

Glycosphingolipids (GSLs) are complex lipids consisting of glycans conjugated to a ceramide core and comprise a diverse group of over 300 molecules (Table 1) (D'Angelo et al., 2013; Nakayama et al., 2013). Although the nomenclature and classification of GSLs is complex (1978), they can be classified on the basis of their electrical charge as neutral, acidic (anionic), or basic (cationic), and according to their core structure as ganglio-series (N-acetylgalactosamine β-1,4 galactose β-1,4 glucose β-1,1′ Cer), globo-series (galactose α-1,4 galactose β-1,4 glucose 1,1′ Cer), isoglobo-series (galactose α-1,3 galactose β-1,4 glucose 1-1′ Cer), muco-series (galactose β-1,4 galactose β1-4 glucose 1,1′ Cer), lacto-series type 1 (galactose β-1,3-N-acetylglucosamine β-1,3 galactose β-1-4 glucose 1-1′ Cer), lacto-series type 2 (galactose β-1,4-N-acetylglucosamine β-1,3 galactose β-1,4 glucose 1-1′ Cer) and gala-series (galactose α-1,4 galactose 1-1′ Cer) (Keusch et al., 2000; Hakomori, 2003; Zhang and Kiechle, 2004; Kopitz, 2017).

**TABLE 1:** Glycolipid types.

GL	Taxon	Lipid backbone	Structure			
Glycoglycerolipids	Eubacteria	Diacylglycerol	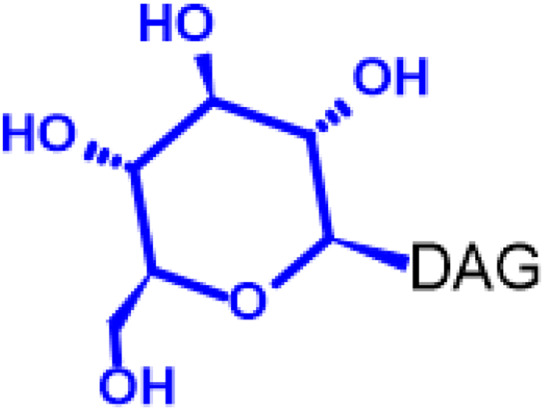			
	Archae	(fatty acid + fatty acid)				
	Plantae	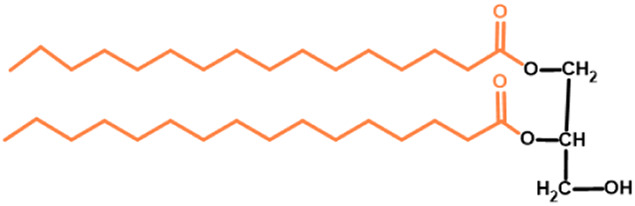				
Glycosylphosphatidylinositol	Animalia	Phosphatidylinositol	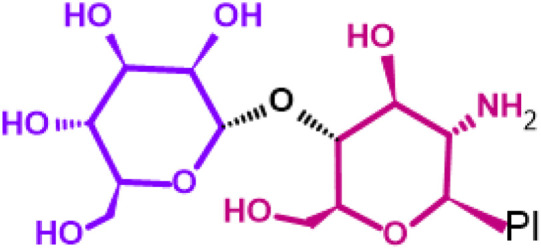			
	Protista	(fatty acid + fatty acid + inositol)				
	Fungi	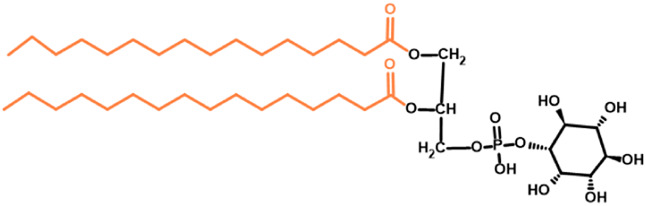				
	Plantae					
Glycosphingolipid	Animalia	Ceramide - Cer-(Sphingosine + fatty acid) 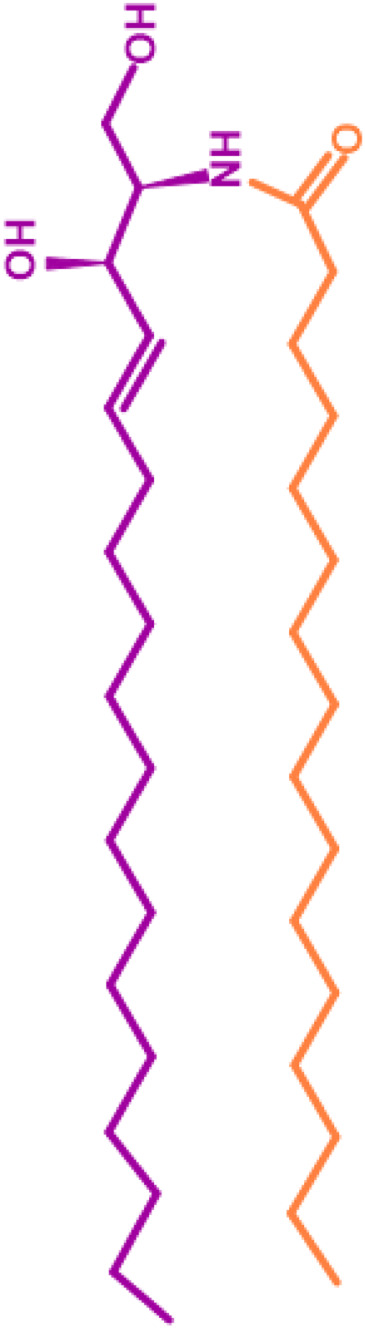	GSL subtypes	Neutral monoglycosylCer	GalCer	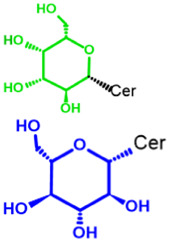
					GlcCer	
				Acid monoglycosylCer	SulfoGalCer	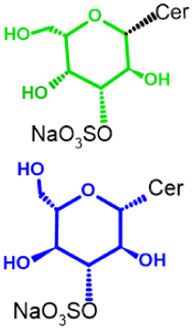
					SulfoGlcCer	
	Protista			Neutral oligoglycosylCer	Gb	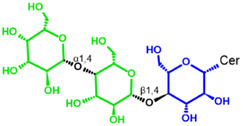
					iGb	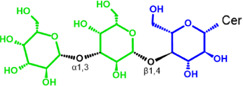
					Mc	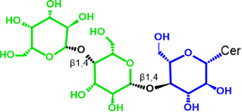
	Fungi				Lc	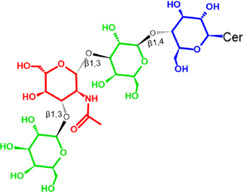
	Plantae				nLc	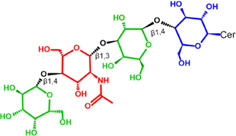
				Acid oligoglycosylCer	Gg	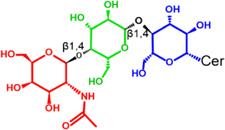
					Ga	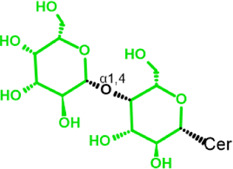

GL, glycolipid; Taxon, taxonomic groups of major occurrences for a GL type; Cer, ceramide; GL structures: blue, glucose (Glc); violet, manose; purple, glucosamine; green, galactose (Gal); red, galactosamine; GSL, glycosphingolipids; Cerebrosides: GalCer, galactosylceramide (left); GlcCer, glucoxylceramide (right); Cerebroside-3-sulfate: SulfoGalCer (left); SulfoGlcCer (right); Gb, globo-series (galactose α-1,4 galactose β-1,4 glucose 1-1′ Cer); iGb, isoglobo-series (galactose α-1,3 galactose β-1,4 glucose 1-1′ Cer); Mc, muco-series (galactose β-1,4 galactose β-1,4 glucose 1-1′ Cer); Lc, lacto-series type 1 (galactose β-1,3-N-acetylglucosamine β-1,3 galactose β-1,4 glucose 1-1′ Cer); nLc, lacto-series type 2 (galactose β-1,4-N-acetylglucosamine β-1,3 galactose β-1,4 glucose 1-1′ Cer); Gg, ganglio-series (N-acetylgalactosamine β-1,4 galactose β-1,4 glucose β-1,1′ Cer); Ga, gala-series (galactose α-1,4 galactose 1-1′ Cer) (Kopitz, 2017).

GSLs are involved in cellular events including the regulation of membrane-receptor protein signaling, cellular crosstalk, cell adhesion and differentiation (Mathow et al., 2015; Herzer et al., 2016; Jennemann et al., 2017). Furthermore, GSLs have been shown to participate in diverse immune processes including differentiation, recognition, recruitment of proteins to specific membrane microdomains, direct interaction with surface receptors, and transduction of activation signals (Zhang et al., 2019). Mice deficient in subclasses of GSLs show immunological, reproductive, neuronal, renal, gastrointestinal, and metabolic defects (Allende and Proia, 2014).

In this context, this review will focus on globotriaosylceramide (Gb3), also known as Burkitt’s lymphoma antigen CD77 (Knapp et al., 1989), and the Pk antigen of red blood cells P antigen system (Naiki and Kato, 1979; Iwamura et al., 2003), a neutral GSL from the globo-series present in a detergent-insoluble portion of lipid raft membranes rich in cholesterol (Fraser et al., 2004). Gb3 is expressed and located in the outer layer of the cell membrane of many different cell types (see Section 3). Although Gb3 is known to fulfill an important role for cell membrane structure in lipid raft microdomains (Allende and Proia, 2014; Mathow et al., 2015; Herzer et al., 2016; Jennemann et al., 2017; Zhang et al., 2019), reports on its biological function remain scarce.

Gb3 is the canonical receptor of Shiga toxin (Stx) from the gram-negative Stx-producing *E. coli* (STEC). Infection with STEC is responsible for hemorrhagic colitis and hemolytic-uremic syndrome (HUS), a thrombotic microangiopathic disease characterized by the triad of microangiopathic hemolytic anemia, thrombocytopenia, and different degrees of acute kidney failure (Karmali et al., 1985). Additionally, the glycosphingolipid catabolism disorder known as Fabry disease causes intracellular deposition of Gb3 in the vascular endothelium and other tissues. This hereditary condition is a heterogeneous, progressive disease whose manifestations include acroparesthesia, sweating abnormalities, cornea verticillata, and angiokeratoma, as well as cardiovascular, cerebrovascular, and renal disorders derived from dysfunction of the organ affected by Gb3 accumulation (Chan and Adam, 2018). Gb3 also serves as an alternative cofactor in CD4-dependent HIV-1 fusion, interacting preferentially with the CXCR4 gp120 (Hammache et al., 1999).

## 2 Gb3 Structure, Synthesis, and Degradation

**FIGURE 1:**
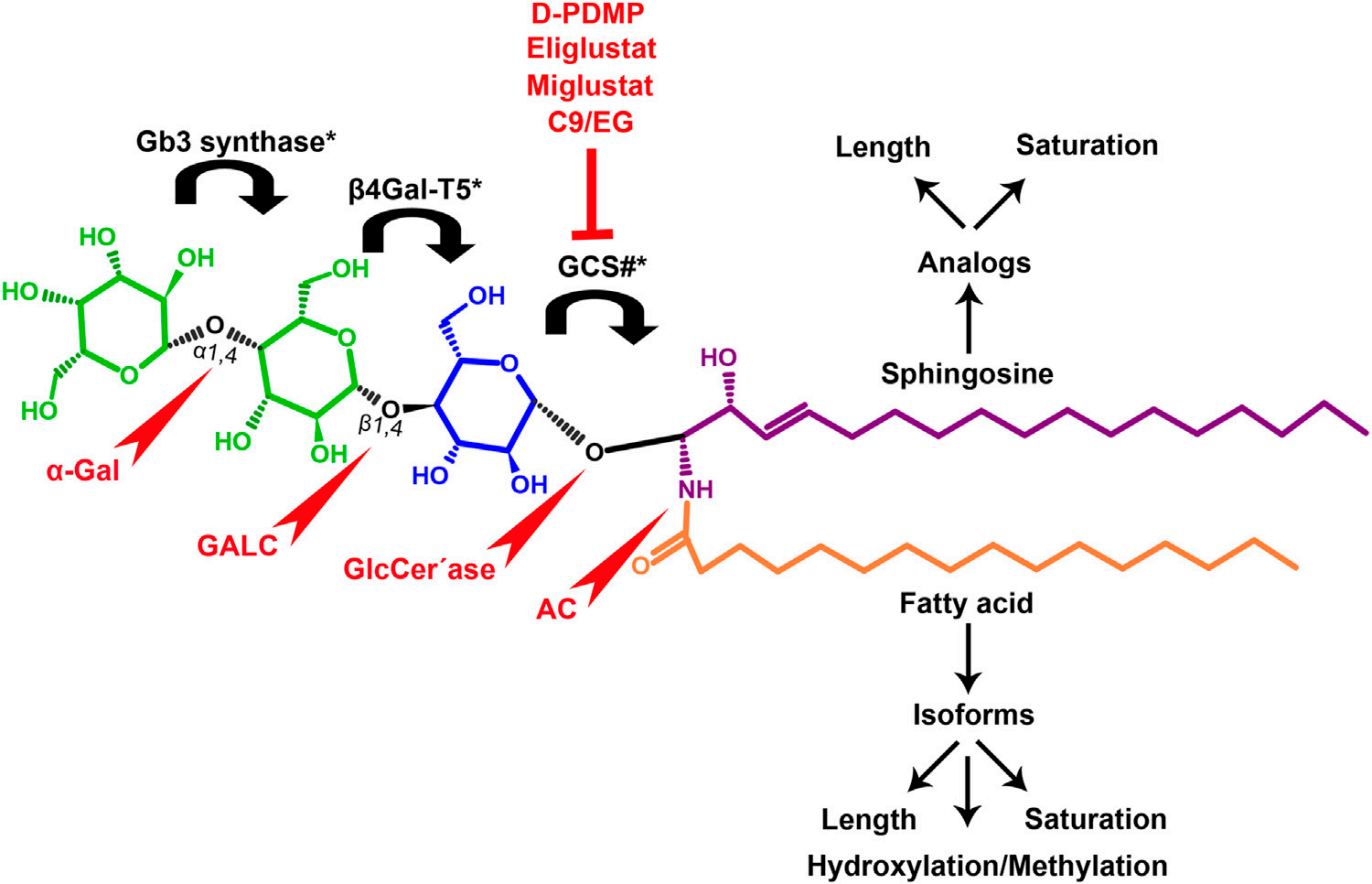
Chemical structure of Gb3. Orange carbon chain: fatty acid. Purple carbon chain: sphingosine. Blue monosaccharide residue: glucose. Green monosaccharide residues: galactose. Black curved arrows represent a specific enzyme in the Gb3 synthesis pathway. The name of the enzyme and the link produced by the enzyme are shown above and below, respectively, in black. Red arrowheads represent a specific enzyme in the Gb3 degradation pathway. The name of the enzyme is shown below in red. Red blunt arrow represents the enzyme which is inhibited by different drugs (above in red) and indicates the changes in the fatty acid and sphingosine that can lead to the diversity of Gb3 isoforms and analogs, respectively. #: Enzyme induced by INFα. *: Enzyme induced by TNFα. GCS, glucosylceramide synthase; β4Gal-T5, β-1,4 galactosyltransferase 5; AC, acid ceramidase; α-Gal, α-galactosidase A; GALC, GalCer-β-galactosidase; GlcCer’ase, GlcCer-β-glucosidase.

Gb3 is a glycosphingolipid formed by a lipid skeleton connected to an oligosaccharide (Figure 1). The lipidic part of its structure consists of the union of a sphingosine and a fatty acid through an amide bond, which renders a ceramide molecule. Gb3 molecules with modifications in the sphingosine are called analogs, while those with modifications in the fatty acid chain are called isoforms (Provencal et al., 2016), the latter mostly accounting for ceramide heterogeneity. Both the fatty acid and sphingosine vary in length, ranging from 16 to 26 carbons. The different Gb3 analogs and isoforms can be classified on the basis of chemical modifications into the following five groups: Group 1, Gb3 isoforms with saturated fatty acids; Group 2, Gb3 isoforms/analogs with an extra double bond (in sphingosine or fatty acid); Group 3, Gb3 isoforms/analogs with two extra double bonds (in the sphingosine and the fatty acid or both in the fatty acid); Group 4, hydroxylated fatty acid Gb3 isoforms; and Group 5, methylated Gb3 isoforms. Of note, these modifications change the affinity of Stx for Gb3 binding (Provencal et al., 2016). Although the oligosaccharide part of Gb3 is the main Stx recognition domain, the lipid fraction can modulate recognition according to its length and degree of unsaturation and hydroxylation (Binnington et al., 2002). These modifications in the lipid part of Gb3 receive support from the detection of different isoforms and analogs of Gb3 in the plasma and urine of patients with Fabry disease which are regarded as biomarkers for this pathology (Provencal et al., 2016).

The synthesis of the ceramide molecule from the sphingosine and the fatty acid takes place in the cytosolic aspects of the endoplasmic reticulum membranes by ceramide synthases (CerS1-6) (Rizzo et al., 2021). The synthesis of the oligosaccharide part of Gb3 takes place in the Golgi apparatus (Budani et al., 2021; Rizzo et al., 2021) and is constituted firstly by the synthesis of a glucosylceramide (GlcCer) produced by the enzyme glucosylceramide synthase (GCS), which catalyzes the binding of a glucose molecule to a ceramide; next, the enzyme β-1,4 galactosyltransferase 5 (β4Gal-T5) binds one molecule of galactose to form lactosylceramide (LacCer); in a third and final reaction, the enzyme lactosylceramide α-1,4-galactosyltransferase (Gb3 synthase) catalyzes the addition of α-1,4 galactose to LacCer (galactose β-1,4 glucose 1-1′ Cer) to produce Gb3 (galactose α-1,4 galactose β-1,4 glucose 1,1′ Cer) (Kojima et al., 2000; Okuda and Nakayama, 2008; Budani et al., 2021; Rizzo et al., 2021). Another enzyme, isoglobotriaosylceramide synthase, can produce polygalactosylated species (Galn-Gb3) by adding α-1,3 galactose residues to Gb3 (Miller et al., 2018). This enzyme also has the ability to act on two further substrates, isoGb3 and LacCer (Keusch et al., 2000).

For Gb3 synthesis, the newly formed ceramide reaches the cis Golgi cisternal through membrane transport, where the cytoplasmic enzyme GCS produces GlcCer in the Golgi cytoplasmic leaflet. Although the translocation of GlcCer to the luminal leaflet and trans Golgi cisternal has not been fully characterized, some studies have reported two possible transport routes: non-vesicular transport and vesicular transport. In non-vesicular trafficking, cytosolic GlcCer is transported by the phosphoinositol-four phosphate adapter protein 2 (FAPP2) from the cis Golgi to trans Golgi and then translocated by an ATP-dependent GlcCer flippase to the luminal leaflet, where the other steps for Gb3 synthesis take place. On the other hand, in vesicular transport (which has been proposed for the synthesis of GSLs of the ganglio-series), the newly synthesized GlcCer in the Golgi luminal leaflet is translocated to the luminal leaflet by an ATP-dependent flippase and delivered to trans Golgi *via* vesicular transport. Regardless of the route taken (vesicular or non-vesicular), once GlcCer reaches the luminal side of the trans Golgi membrane, it becomes the substrate of β4Gal-T5 with the formation of LacCer, a precursor for the synthesis of globo-, ganglio-, lacto- and neolacto-series of GSLs (Budani et al., 2021; Rizzo et al., 2021).

Other proteins responsible for GlcCer transport through cis-trans Golgi are members of the glycolipid transfer protein superfamily (GLTP). Although GLTP is considered homologous to FAPP2, its transport route remains mostly unknown. However, *in vitro* studies have shown that the synthesis of GLSs is affected by a reduction in FAPP2 but, surprisingly, not affected by a reduction in GLTP (Kjellberg and Mattjus, 2013).

Contrary to what occurs in the synthesis of protein, RNA and DNA, the synthesis of GSLs proceeds in a template-independent manner and only depends on the order in which glycosyltransferases add specific monosaccharide residues to the growing glycan chain in the ceramide molecule through competing reactions taking place in the cisternae of the Golgi apparatus (D'Angelo et al., 2013). Each of the Golgi cisternae (cis to trans) contains different glycosyltransferases acting sequentially in the synthesis of the different GSLs. Therefore, the intra-Golgi distribution of each glycosyltransferase determines the composition of the glycan chain and the type of GSL synthesized (Papanikou and Glick, 2014). Therefore, the mechanism controlling intra-Golgi transport and the final position of GSL synthetic enzymes are key determinants of the final GSL produced by each cell (Palm, 2021).

GOLPH3 and GRASP55 are some of the proteins responsible for such mechanism. GOLPH3 recognizes Golgi-resident enzymes and mediates their sorting into coat complex protein I (COPI) vesicles for retrograde transport, thus producing the recycling of enzymes in each cisterna. In other words, GOLPH3 works as a COPI adapter while recognizing specific domains of glycosylation enzymes present in the Golgi such as LCS. Thus, by changing GOLPH3 levels, cells can adjust the fraction of enzymes retained in the Golgi and not sent to lysosomal degradation (Palm, 2021; Rizzo et al., 2021). Otherwise, Golgi matrix protein GRASP55 prevents the retrograde transport of glycosyltransferases, retaining these enzymes in the trans Golgi. Therefore, a balance between the actions of GOLPH3 and GRASP55 determines the localization and levels of intra-Golgi glycosyltransferases (Pothukuchi et al., 2021).

Several other proteins whose roles remain to be elucidated are responsible for the intra-Golgi transport of GSL synthetic enzymes. Although a detailed discussion of all known processes and proteins responsible for this mechanism is beyond the scope of the present review, it is worth mentioning that the transmembrane protein 165 (TMEM165), the transmembrane 9 superfamily member 2 (TM9SF2) and LAPTM4A proteins localized within the Golgi apparatus modulate the activity, localization, and transport of GSL biosynthetic factors, including Gb3 (Pacheco et al., 2018; Tian et al., 2018; Yamaji et al., 2019).

Conversely, Gb3 degradation occurs in lysosomes and proceeds in four steps by the action of acid hydrolases which consecutively eliminate each of the carbohydrate residues from Gb3. The process ends with the elimination of the fatty acid chain from the ceramide, thus producing three free hexoses, a free chain of free fatty acids, as well as a sphingosine. More specifically, the four steps take place as follows: 1) an α-galactosidase A (α-Gal) removes the terminal α-galactose residues from Gb3 to remain as LacCer; 2) the GalCer-β-galactosidase (GALC) removes the remain terminal β-galactose residue from LacCer to yield GlcCer; and 3) the GlcCer-β-glucosidase (GlcCer’ase) removes the remain terminal glucose from the ceramide. Finally, 4) an acid ceramidase (AC) removes the fatty acid chain from the ceramide to form sphingosine (Miller et al., 2018; Breiden and Sandhoff, 2019). Deficiencies in the enzymes responsible for degradation lead to diseases such as Fabry, Gaucher, Sandhoffand, and Tay Sach, which are characterized by the lysosomal accumulation of GSLs, including Gb3, in different cell types (Abe et al., 2000; McEachern et al., 2007).

Several studies have indicated that the use of specific inhibitors of GSL synthesis could mitigate the pathological phenotype. The group led by J Müthing used a ceramide analog, D-threo-1-phenyl-2-decanoylamino-3-morpholino-1-propanol (D-PDMP), as an inhibitor of GLS biosynthesis. D-PDMP is frequently used to study GSLs in biological processes such as cell signaling, cell growth, and cell differentiation, as well as in studies of protein-carbohydrate interactions and host bacteria (Abe et al., 2000; Shayman et al., 2000; Legros et al., 2017b). D-PDMP treatments in kidney proximal tubular cells and human glomerular endothelial cells (HGECs) revealed a decrease in GLS synthesis not only from GlcCer and Lc2Cer but also from Gb3 and Gb4 (Legros et al., 2017b; Sanchez et al., 2021). Silberstein et al. (2011) have shown that C-9 (Genzyme, Waltham, MA) is a specific inhibitor of GCS which reduces the synthesis of Gb3 and could therefore be used as a therapeutic agent for the diseases mentioned above. In addition, the cytotoxic effects of Stx are mitigated by this inhibitor in primary cultures of human renal tubular epithelium cells (HRTEC), which makes it a potential treatment for patients with HUS (Silberstein et al., 2011). Moreover, Sanchez et al. (2021) have observed that Eliglustat (EG), a potent and selective inhibitor of GCS, provides protection against the action of Stx on cell proliferation and apoptosis/necrosis mechanisms in both HRTEC and the human proximal tubule cell line HK2. Although both EG and C-9 could be used to prevent human kidney damage caused by Stx, EG requires a lower dose and incubation time to inhibit Gb3 expression and achieve total protection against Stx *in vitro*. Worth highlighting, EG has been recently approved by the National Administration of Medicines, Food and Medical Devices (ANMAT) of Argentina to be used in oral treatment for adults with Gaucher disease (Sanchez et al., 2021).

Extensive evidence shows that GSL synthesis could be regulated by cytokines such as IFNα, TNFα and IL-1: IFNα increases the expression of genes involved in Gb3 regulation, which results in unbalanced accumulation. For instance, IFNα induces GCS gene expression, increasing LacCer and GlcCer Gb3 precursors (Tan et al., 2018). Moon et al. (2005) observed that during the development of HUS, TNFα and IL-1 expressions are induced by Stx in association with LPS, damaging endothelial and intestinal epithelial cells. *In vitro* studies performed on human HT29 colon epithelial cells have shown that TNFα increases the synthesis of Gb3 through GCS, and upregulates Beta4Gal-T5 and Gb3 through NF-kappaB signaling in mitogen-activated protein kinase (p38, JNK 1/2 and ERK 1/2)-induced cascades (Moon et al., 2005).

## 3 Gb3 Localization

Gb3 is present on the cell membrane of a wide variety of mammalian tissue cells. Its expression levels have been associated with pathological or physiological states, depending on whether its canonical ligand Stx participates or not. Furthermore, as with other glycosphingolipids, the presence and quantity of Gb3 on the membrane of blood cells has been used as an indicator of cell maturation state and lineage. This section will discuss the different cell types that express Gb3 and the associated findings (Table 2).

**TABLE 2:** Overview of Gb3 allocation and its physiological and/or pathological functional alternatives.

Sp.	Organ/Tissue/Cell type	Physiological function	Pathological role	References
Mo 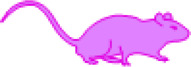	Lymphoid lineage	degree of activation (↑, activated lymphocyte)		Stein and Marcus (1977); Kiguchi et al. (1990); Schwartz-Albiez et al. (1990); Madassery et al. (1991)
	Monocyte lineage	membrane differentiation marker (↓, MDP; ↑, MM)		Kniep et al. (1985)
	Bone marrow/dendritic and mast cells	DCs differentiation inducer⇒ NKT cells activation		Li et al. (2009); Katz et al., (1985)
		mast cells activation marker		
	Kidney/proximal tubule	membrane stabilizer⇒ albumin reabsorption and proteins filtered	⇒proteinuria and albuminuria	Morace et al. (2019)
	PNS/DRG/sensory neurons	NA	NA	Ren et al. (1999)
	CNS/neurons	NA	Stx-induced: ↑ Gb3; glutamate release and neurodegeneration	Obata et al. (2008); Obata and Obring. (2010)
	CNS/endothelial cells	NA		Fujii et al. (1994); Obata et al. (2008); Pinto et al. (2013)
	CNS/astrocytes	⇒cell growth and differentiation	Stx-induced apoptosis	Majoul et al. (2002)
Hu 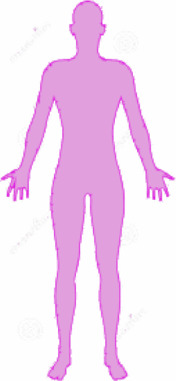	Lung/epithelium		NA	Uchida et al. (1999)
	Kidney/glomerular endothelium/tubular epithelium proximal, distal and collecting ducts/podocytes and mesangial cells		Stx-induced cell death	Karpman et al. (1998)
	Erythrocytes	Blood phenotype		Yang et al. (1994); Naiki and Kato. (1979)
	/megakaryocytes	NA		Furukawa et al. (2002)
	Lymphoid organs/GC/B and T cells	antigen-driven selection		Mangeney et al. (1991); Pascual et al. (1994)
	Lymphoid organs/tonsillar lymph node/lymphocytes	NA		Stein and Marcus (1977); Kiguchi et al. (1990); Schwartz-Albiez et al. (1990)
	CNS/neurons and endothelial cells	NA	NA	Obata and Obring. (2010)
	CNS/astrocyte		↑IL-1ß	Kioka. et al. (2012)
	PNS/DRG/sensory neurons and endothelial cells	NA		Obata and Obring. (2010)
Ra 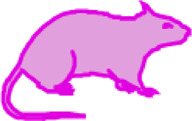	CNS/neurons		Stx-induced: ↑ Gb3 and neurodegeneration	Tironi-Farinati. et al. (2010)
	CNS/microglial cells		Stx-triggered microglial activation and neuronal damage	Berdasco et al. (2019)
	CNS/astrocytes		Stx-triggered astrocytic reactivity and ↑Gb3	Tironi-Farinati et al. (2010)
	PNS/DRG/sensory neurons	NA		Obata and Obring. (2010)
	Kidney		Stx-induced injury	Silberstein et al. (2011)
Rb 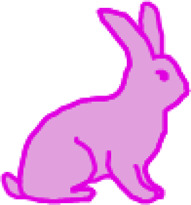	Intestinal epithelium/MVM		Stx cytotoxicity	Mobassaleh et al. (1988)
	CNS/parenchyma/endothelial cells		⇒ microglial activation and neuronal damage	Takahashi et al. (2008)
			Endothelial injury and microvascular thrombosis	
	PNS/DRG/sensory neurons and endothelial cells	NA		Obata and Obring. (2010)
	Kidney		Stx-induced injury	Silberstein et al. (2011); Garcia et al. (2006)
Cell lines 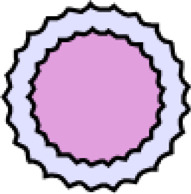	Burkitt lymphoma	CD19 interaction ⇒ B cells differentiation	Stx cytotoxicity: protein synthesis inhibition	Maloney and Lingwood. (1994)
	Vero, Caco-2 and Hep-2 cells	⇒Cellular migration	apoptosis/necrosis triggering	Mannori et al. (1990); Karpman et al. (1998)
	HRTEC, HK2		Stx⇒death-sensitive	Sanchez et al. (2021); Jones et al., (2000)
	COS-7	FAS pathway ⇒ cell death signaling activation		Chakrabandhu et al. (2008)
	HBECs		apoptosis/necrosis triggering	Ergonul et al., (2003); Takahashi et al. (2008)
	HUVECs			Molostvav et al. (2001)

Sp., animal model; Mo, mouse; Hu, human; Ra, rat; Rb, rabbit; ↑, increased expression level; ↓, decreased expression level; MDP, monocyte precursor; MM, mature monocyte; DCs, dendritic cells; ⇒, associated with; NKT, Natural killer T cells; PNS, peripheral nervous system; DRG, dorsal root ganglia; NA, not available; CNS, central nervous system; Stx, Shiga toxin; GC, germinal center; MVM, intestinal microvillus membrane; HRTEC, human renal tubular epithelium cells; HK2, human proximal tubule; HBECs, human brain microvascular endothelial cells.

### 3.1 *In Vivo* Gb3 Localization

The expression and localization of Gb3 may vary across different species, which calls for caution in translating experimental conclusions from animal models to humans. Gb3 is expressed in the glomerular endothelium, podocytes, tubular epithelia, and mesangial cells in the human kidney, but it is only expressed in the proximal tubular epithelium in mice (Silberstein et al., 2011; Morace et al., 2019). Renal expression of Gb3 in rats and rabbits makes them suitable models of STEC infection to study kidney lesions similar to those caused during HUS in humans (Garcia et al., 2006; Silberstein et al., 2011).

In the gut, the presence of Gb3 has been described in rabbit intestinal microvillus membrane (MVM), as shown using radioactively labeled Stx in ligated intestinal loops. In these experiments, Gb3 was identified as the receptor of Stx in the MVM. Furthermore, this receptor increases after 16 days of life in rabbits, thus increasing cytotoxicity in the MVM model (Mobassaleh et al., 1988).

In the peripheral nervous system (PNS) and the central nervous system (CNS), it is noteworthy that Gb3 expression varies in neural cells both across species and depending on whether it is induced by Stx or not. In the PNS, Gb3 has been found in human and rabbit dorsal root ganglia (DRG) neurons and endothelial cells, whereas it is exclusively localized in neurons in mice and rats (Obata, 2010). In turn, in the CNS, Gb3 has been detected in neurons and endothelial cells of human cadavers, but in murines it was only detected in neurons (Obata, 2010). Localization discrepancies have also been reported: Okuda et al. (2006) postulate Gb3 expression in murine endothelial cells and not in neurons (Okuda et al., 2006); in contrast, Kolling et al. (2008), who observed Gb3 in murine neurons and not in endothelial cells, argue that differences may respond to tissue fixation procedures. In other words, Okuda and collaborators used immersion fixation, which produces false positive immunolabeling, while Kolling et al. carried out perfusion fixation (Kolling et al., 2008).

The expression of Gb3 has been detected in rabbit brain endothelial cells with no Stx induction through direct immunofluorescence using a Gb3 antibody (Ren et al., 1999; Utsunomiya et al., 2001; Takahashi et al., 2008). Although Gb3 has also been immunodetected in endothelial cells upon Stx induction using an antibody against Stx, which in turn binds the Gb3 receptor (Richardson et al., 1992; Fujii et al., 1996; Mizuguchi et al., 1996), this indirect method seems less accurate than direct Gb3 receptor detection. In addition, non-Stx-induced Gb3 has been observed in humans and mice (Ren et al., 1999; Nishikawa et al., 2002), while Stx-induced Gb3 has been reported in mice through the use of frozen sections fixed with ice-cold acetone (Okuda et al., 2006).

Gb3 expression has been found in brain neurons of rats both treated and non-treated with Stx, fixed by perfusion and subjected to anti-Gb3 immunodetection (Tironi-Farinati et al., 2010). In mice, immunodetection has been obtained by immunoelectron microscopy using an indirect method with an anti-Stx2 antibody in Stx-treated animals (Fujii et al., 1994), in formalin-fixed brains indirectly immunodetected with an anti-Stx antibody (Obata et al., 2008), and in non-treated brains fixed by perfusion (Obata et al., 2008; Obata and Obrig, 2010).

The relevance of pro-inflammatory cytokines in CNS damage initiated by Stx2 has been demonstrated in rabbits, where microglial activation and overexpression of TNFα are associated with the expression of Gb3 in the endothelium (Takahashi et al., 2008). However, the presence of Gb3 in the brain is not exclusive to endothelial cells. Obata. (2010) and Tironi-Farinati et al. (2010) have shown the neuronal presence of this receptor in mice and rats, respectively (Obata, 2010; Tironi-Farinati et al., 2010). Furthermore, Tironi-Farinati et al. (2010) have shown increased Stx2 receptor expression for the first time in neurons of the striatum, hippocampus and cortex, and in reactive astrocytic processes surrounding neurons (Tironi-Farinati et al., 2010). Later on, the induction of Gb3 by Stx2 was demonstrated in a murine model. Results showed that anti-inflammatory compounds like dexamethasone reduce Gb3 expression in neurons, highlighting the pivotal role of inflammation processes in encephalopathy produced by STEC (Pinto et al., 2013; Pinto et al., 2017). The Gb3 receptor has also been reported in organs other than kidney and brain, for instance, in the lung epithelium and vasculature of patients suffering from HUS (Uchida et al., 1999).

### 3.2 *In Vitro* Gb3 Localization

Regarding the CNS, Gb3 has been found in brain microvascular endothelial culture cells (HBEC), which are death-sensitive following Stx incubation (Ergonul et al., 2003; Takahashi et al., 2008). Accordingly, pro-inflammatory cytokines such as TNFα and/or IL-1β markedly increase the content of Gb3 and the capacity of Stx binding in HBEC, which results in apoptotic cell death.

Gb3 expression has also been reported through immunohistochemistry in astrocyte cultures obtained from human fetal brain tissue. Incubation with IL-1β increased Gb3 expression as well as Gb3 synthase, involved in Gb3 synthesis. However, micrographs showing Gb3 immunohistochemistry were captured in low magnification and no quantification or corresponding controls were carried out, which makes Gb3 expression in these astrocyte cultures uncertain (Kioka et al., 2012).

The presence of Gb3 has also been reported in cultures of primary fetal mouse astrocytes. In this case, Gb3 expression was immunodetected on the cell surface and varied according to the state of the cell cycle, peaking at G2 and metaphase phases. Although these findings may hint at Gb3 involvement in the tuning of cell growth and differentiation, the study was carried out using Stx as a Gb3 ligand, which renders indirect determination of Gb3 and fails to rule out Stx binding to other non-canonical receptors (Majoul et al., 2002).

In rat microglial primary cultures, Stx2 is incorporated through Gb3-dependent and independent pathways. Recent work by Berdasco et al. (2019) confirmed the presence of Gb3 in microglia primary cultures and showed that these cells have context-specific reactions to Stx2 which included either a fever condition or the presence of endotoxin upon treatment with Stx2. The metabolic and functional states, as well as the subcellular distribution and expression of Gb3, vary according to the context, promoting the expression of pro-inflammatory cytokines and microglial phagocytic capacity (Berdasco et al., 2019). In addition, in Vero, Burkitt’s lymphoma, Caco-2 and Hep-2 cell lines, the binding of Stx to Gb3 induces cytotoxic effects by inhibiting protein synthesis and inducing apoptosis and necrosis (Karpman et al., 1998; Jones et al., 2000; Silberstein et al., 2011).

#### 3.2.1 Gb3 in Hematopoietic Cells

Due to their immunogenicity, GSLs have been used to differentiate human cell lines and establish differentiation stages. In particular, Kniep et al. (1985) used them to identify the stages of myeloid cell differentiation and reported that GSLs of the globo-series are characteristic of the monocytic lineage, with Gb3 and Gb4 expression increasing as monocytic precursors mature (Kniep et al., 1985).

Gb3, isoGb3 and Gb4 have also been identified as some of the most abundant molecules in dendritic cells differentiated *in vitro* from mouse bone marrow progenitor cells. Furthermore, evidence suggests that isoGb3 from these cells may act as an activator of NKT cells (Li et al., 2009).

Gb3 has been reported as one of the major GLSs in human blood platelets (Cooling et al., 1998). The presence of this GLS on the membrane derives from its expression in megakaryocytes, which subsequently fragment to give platelets (Furukawa et al., 2002).

Mouse bone marrow cells can also differentiate into mast cells *in vitro* and exhibit GSLs of the globoside family on their membrane. In a state of activation, these globosides increase their concentration due to exocytosis by globoside-rich granulocytes (Katz et al., 1985).

In human erythrocytes, glycolipids are antigenic determinants in different blood group phenotypes. These glycolipids are chemically based on the sequence Galα4Gal and comprise a system of P antigens (i.e., PK and LKE) and five phenotypes (i.e., P1, P2, P1K, P2K, p) (Naiki and Kato, 1979). The PK antigen is found in small amounts in red blood cells from individuals with the P1 and P2 phenotypes but enriched in the P1k and P2k phenotypes (Yang et al., 1994).

The presence of Gb3 in lymphocytes has been described by Stein since the early 1970s. This work reports a larger proportion of Gb3 in human lymphocytes from the tonsilla than in peripheral blood or thymic lymphocytes (Stein and Marcus, 1977). Moreover, Kiguchi et al. (1990) have shown that, within the human leukocyte lineage (lymphocytes, monocytes and granulocytes), 4% triacyl glycolipids correspond to Gb3 expressed only in lymphocyte membranes (Kiguchi et al., 1990). Further studies have shown only traces of Gb3 in peripheral blood lymphocytes and thymocytes (Schwartz-Albiez et al., 1990). Later, Madassery et al. (1991) showed a more prominent presence of Gb3 in activated murine and human lymphocyte extracts *in vitro* (Madassery et al., 1991). Altogether, these studies conclude that Gb3 is mainly present in B lymphocytes and that its expression is associated with the degree of activation in the germinal centers.

## 4 Physiological Function of Gb3

Changes in GSLs composition patterns are critical in neural differentiation. It has been previously determined that human embryonic stem cells rich in the globo-series GSLs differentiate into neural cells rich in ganglio-series (Liang et al., 2010, 2011). Later, Gb3 was shown to specifically inhibit the expression of neural markers in murine embryonic stem cells, which then failed to differentiate into either neural or glial populations (Russo et al., 2018). Gb3 reduces upregulated GM3S (GM3 synthase, a key regulator of ganglioside GSL production), which indicates that globo-series GSLs prevent neural differentiation. Specifically, Gb3 negatively regulates the expression of the autism susceptibility gene AUTS2, a master epigenetic modulator of neuronal differentiation. AUTS2 binds the GM3S promoter site involved in the expression GM3S, causing the switch to the ganglio-series GSL expression and allowing neuronal differentiation. Therefore, a globo-AUTS2 axis may be thought to control the expression of genes involved in neurogenesis, a relevant finding given that mutations in AUTS2 and GM3S genes provoke severe clinical neural defects (Beunders et al., 2013).

It was long thought that Gb3 was specifically expressed by germinal center B cells undergoing apoptosis. At first, this was hypothesized as a mechanism for the elimination of cells which did not produce high-affinity antibodies during antigen-driven selection in the germinal center of secondary lymphatic organs (Mangeney et al., 1991). However, it was later suggested that Gb3 was expressed in a germinal center B cell subset engaged in the somatic hypermutation of immunoglobulin (Ig) genes called centroblasts (IgD-/CD38+/CD77+), and that these cells did not undergo apoptosis (Pascual et al., 1994). CD77 was then used to discriminate between two germinal center B cell subsets, centroblasts (IgD-/CD38+/CD77+) and centrocytes (IgD-/CD38+/CD77-), the latter engaged in Ig class switch recombination processes. More recently, it was observed that these two groups of B cells, which initially differentiate through the presence or absence of Gb3, show an equally active DNA repair program, as well as components involved in somatic hypermutation and Ig class switch recombination. Therefore, the discrimination of centroblasts and centrocytes by the expression of Gb3 and the role of Gb3 in the induction of apoptosis remain controversial (Klein et al., 2003; Hogerkorp and Borrebaeck, 2006).

A similar finding regarding the expression of Gb3 by germinal center B cells undergoing apoptosis was obtained in human umbilical vein endothelial cells (HUVECs) (Molostvov et al., 2001), which were found to express almost undetectable levels of Gb3. However, a direct relationship was observed between the expression of Gb3 and apoptosis in these cells. Interestingly, apoptosis was independent of the action of Stx, as HUVEC incubation with a combination of TNFα and IFNγ or endothelial cell growth factor (ECGF) deprivation produced a significant increase in Gb3 expression accompanied by apoptosis. Nevertheless, it is still unclear whether the expression of Gb3 is necessary for or is the direct cause of apoptosis. Moreover, the association between Gb3 expression and apoptosis in germinal center B cells and HUVECs may be thought of as two distinct phenomena having no correlation. Alternatively, paracrine production of an unknown Gb3 ligand may be responsible for triggering apoptosis in these cells.

The quality and strength of the signal transduced through the B-cell antigen receptor (BCR) can be significantly modulated by co-receptor molecules such as CD19, a B cell transmembrane glycoprotein and a member of the Ig superfamily (Del Nagro et al., 2005). In mature B cells, CD19 forms a multimolecular signal transduction complex with CD21, CD81, and Leu-13 (Tedder et al., 1994). The extracellular domain of CD19 has an extensive sequence similarity to StxB subunit (see Section 5.1), with a potential site of Gb3 interaction. Burkitt’s lymphoma-derived cells–an experimental model of B cells from the germinal center–bind matrices containing terminal Gb3 galactose α-1,4 galactose through CD19. This binding is blocked by matrix pretreatment with StxB, anti-Gb3 monoclonal antibody (mAb), or anti-CD19 mAb. As expected, these cells also present homotypic adhesion mediated by the binding of CD19 to Gb3 molecules on adjacent cells, in a manner analogous to Burkitt’s lymphoma-derived cells CD19/matrix Gb3 binding (Maloney and Lingwood, 1994).

In contrast, Gb3-deficient mutant cells with low surface expression of CD19 neither present homotypic adhesion nor bind matrices containing terminal Gb3 galactose α-1,4 galactose. However, these cells do exhibit conserved expression of other B cell markers such as CD10, CD20, HLA-DR, and IgM, which suggests that Gb3 expression influences the surface expression of CD19. It has been postulated that CD19 and Gb3 associate on the B cell surface, and that Gb3 could thus be considered a component of the CD19 complex in Burkitt’s lymphoma and germinal center B cells, together with other proteins such as CD21 and TAPA-1. It is possible that the presence of Gb3 changes the conformation of CD19, thereby causing an increase in antibody recognition (Maloney and Lingwood, 1994).

Gb3 was also found to be important for the FAS receptor-ligand system (Chakrabandhu et al., 2008), one of the main mechanisms to eliminate germ-infected cells and to control autoimmune disease and certain malignancies (Fas et al., 2006). The cell surface-bound receptor FAS belongs to the TNF receptor family containing an intra-cellular death domain which triggers apoptosis (Yamada et al., 2017). Its physiological ligand, FASL, is a member of the corresponding TNF cytokine family expressed on the surface of classical CD8+ cytotoxic T cells, as well as natural killer T cells. However, FAS internalization is a requisite for the activation of apoptotic pathways, whereas its non-internalization results in the activation of nonapoptotic signaling pathways leading to proliferative Erk and NF-kappaB signaling (Lee et al., 2006). FAS has a conserved extracellular Gb3 binding motif—it also interacts strongly with lactosylceramide and weakly with disialoganglioside GD3 and Gb4—which regulates its internalization route and consequently leads to clathrin-mediated FAS internalization and the subsequent transduction of cell death signals. Therefore, the presence of Gb3 interacting with the conserved extracellular Gb3 binding motif is essential for the activation of the FAS apoptotic pathway (Chakrabandhu et al., 2008).

Another protection mechanism capable of inducing an antiviral state in both virus-infected and uninfected cells, and in which Gb3 was found to be relevant, is the type I interferon (INF) system (Yan and Chen, 2012). The type I INF family is a multi-gene cytokine family in which INFα and β are the best-defined and most broadly expressed type I IFNs (Pestka et al., 2004). Almost all body cells can produce and respond to IFNα/β, and its expression occurs in response to the stimulation of pattern recognition receptors by microbial products (McNab et al., 2015). However, even if IFNα2 can bind to its receptor in many cell types (Lau et al., 1986), ability to respond to this cytokine is limited to few cell types *in vitro* (Hannigan et al., 1984). IFNα2-responding cells are also interestingly sensitive to the deleterious effects of Stx (Cohen et al., 1987). It was first observed that transformed ganglioside-deficient mouse cell lines were relatively insensitive to INFα action (Vengris et al., 1976). It was later shown using the Daudi lymphoma cell line, which is highly susceptible to Stx cytotoxicity and INFα2 action, that mutant cells which did not express Gb3 were highly resistant to INFa2 antiviral action, although INFa2 produced growth inhibition (Cohen et al., 1987).

Type 1 IFN receptor (IFNAR) is a heterodimeric transmembrane receptor composed of subunits IFNAR1 and IFNAR2. Canonically, when type I INF binds to IFNAR, it activates the receptor-associated protein tyrosine kinases Janus kinase 1 (JAK1) and tyrosine kinase 2 (TYK2). This event leads to the phosphorylation of the latent cytoplasmic transcription factor signal transducer and activator of transcription 1 (STAT1) and STAT2, which results in the expression of different genes in a cell type- and context-dependent manner (Ivashkiv and Donlin, 2014). Surface expression of IFNAR1 is not Gb3-dependent, although lateral association between Gb3 and IFNAR1 is required for IFNAR1-mediated signal transduction and antiviral activity (Khine and Lingwood, 2000). A comparison between the amino acid sequences of IFNAR1 and Stx shows three closely spaced regions of similarity between the B-subunit of Stx and the IFNα2 receptor, which suggests that these similar sequences may lead to specific interaction between Gb3 and IFNAR within the plasma membrane. This interaction may in turn result in the allosteric generation of the higher-affinity α2-interferon-binding site, which has been implicated in the mediation of this cytokine biological activity (Lingwood and Yiu, 1992). Furthermore, the length of the chain fatty acid from Gb3 isoforms is also relevant for this interaction and subsequent receptor internalization. Cells with predominantly long chain fatty acid Gb3 isoforms are more sensitive to IFNα2-mediated antiviral activity than cells with predominantly short chain fatty acid Gb3 isoforms and receptor internalization in the Golgi apparatus. Conversely, short chain fatty acid Gb3 isoforms are more sensitive to IFNα2-mediated inhibition of proliferation and show receptor internalization in the endoplasmic reticulum/nucleus (Arab and Lingwood, 1998; Khine and Lingwood, 2000).

A StxB-like amino acid sequence has also been described in the beta-chain of human and murine human leukocyte antigen (HLA) and major histocompatibility complex (MHC) class II molecules, respectively, which indicates that this sequence has a potential site of Gb3 interaction with these two analogous molecules (George et al., 2001). B cells, along with dendritic cells and macrophages, are considered professional antigen-presenting cells, as they all express MHC class II molecules and may thus present exogenous antigens to T CD4+ cells. Besides expressing MHC class II molecules, these three professional antigen-presenting cells also express Gb3 (Mangeney et al., 1991; Ramegowda and Tesh, 1996; Lee et al., 1998). It has been suggested that Gb3 binding in this site could modulate the peptide-binding properties of these MHC class II molecules. Furthermore, the presence of Gb3 in professional antigen-presenting cells also seems to modulate HLA expression, as Burkitt’s lymphoma-derived cells exhibit higher levels of HLA than Gb3-deficient mutant cells. However, surface expression of HLA is not regulated by Gb3, as evidenced by equivalent levels of HLA in Gb3-expressing and Gb3-deficient cells (George et al., 2001).

However, it is not yet clear whether all these proposed immunological functions of Gb3, which were observed in *in vitro* models, are truly relevant to or correlate with real *in vivo* immunological functions. Phase 1 pharmacokinetics, safety, and tolerability studies of two clinically approved and used drugs for the treatment of Fabry and Gaucher disease, Eliglustat and Miglustat, are both inhibitors of GCS and have shown no adverse immunological effects (Maegawa et al., 2009; Peterschmitt et al., 2011), which is in keeping with the results described above. This suggests that there may be no correlation between *in vitro* and *in vivo* findings on Gb3 in the action of CD19, HLA, FAS and type I IFN system, or else that still unknown compensatory mechanisms may offset the absence of Gb3.

At variance, a relevant function of Gb3 has been recently found in kidney proximal tubular epithelial cells (Morace et al., 2019). Using a Gb3 deficiency murine model (knockout of Gb3-synthase), this study showed that Gb3-deficient mice had a significant increase in urinary albumin and low molecular weight protein, which indicates that Gb3 plays a relevant role in albumin reabsorption and low molecular weight protein filtering into primary urine. However, this action seems to be related to the presence of Gb3 in lipid rafts and, therefore, to membrane stability in epithelial cells of the proximal tubules, rather than to a direct participation of Gb3 as a receptor in a ligand-receptor-type mechanism.

### 4.1 Gb3-Bound Ligands

Before considering Gb3-binding ligands, it is worth briefly revisiting GSL structure, in which a ceramide (Merrill, 2011) is the basic lipid backbone. Glycosidic linkages attach monosaccharides or oligosaccharide chains, determining monoglycosylceramides and oligoglycosylceramides, respectively. Globoceramides (Gb) are examples of neutral oligoglycosylceramides, while gangliosides are examples of acidic oligoglycosylceramides. Their structural features allow GSLs to spontaneously form lipid rafts in biological membranes (Prinetti et al., 2009; Lingwood and Simons, 2010; Honigmann and Pralle, 2016) and thus significantly influence their topological aspects. This lipid microdomain (LiMd) concept, coupled with the idea that biological functions are also coded by cell surface glycan structures, may promote a wider knowledge of cell biology, physiological (see Section 6.2) as well as pathological.

GSLs canonical role as pathogen (Karlsson, 1989; Matrosovich et al., 2015) and bacterial toxin (Fantini et al., 2000; Binz and Rummel, 2009; Schuller, 2011; Muanprasat and Chatsudthipong, 2013; Legros et al., 2017a) binding sites on the outer cell membrane leaflet have been reported, and these attachments have been shown to trigger a lipid-raft-mediated endocytic degradation pathway (Ewers and Helenius, 2011). Many *in vitro* studies have proven that different Gb3 ligands could trigger apoptotic signals in two different ways, i.e., a caspase-dependent manner and a ROS-dependent one. Stx1-induced apoptosis involves caspase activation. Moreover, it has been postulated that Stx1-induced apoptosis in Burkitt’s lymphoma cells proceeds through the ubiquitin–proteasome pathway triggered by caspase-8 inhibitory molecule (c-FLIPL) degradation (Devenica et al., 2011). Anti-Gb3 antibody induces the ROS-dependent pathway, and this oxidative stress seems to mediate cell death mainly due to 1) cleavage and activation of the pro-apoptotic Bcl-2 family member Bid, and 2) Bax relocalization to mitochondrial membranes, which leads to cytochrome c release (Devenica et al., 2011). Studies conducted on THP1 myelogenous leukemia and epithelial cell lines have shown how Stx induces an apoptotic pathway dependent on both caspases and ROS production (Lee et al., 2008). Researchers found out that apoptosis in endothelial cells occurs due to toxin inhibition of Mcl-1 expression, an anti-apoptotic member of the Bcl-2 family (Erwert et al., 2003). Interestingly, in HeLa cells, Stx induces apoptosis *via* an extrinsic pathway through the activation of caspase-8, 6 and 3 (Fujii et al., 2003).

Despite the evidence of Gb3 activation of apoptotic pathways discussed above, no direct endogenous ligands have been found so far. Conceivably, *in vivo* physiological ligand identification may remain elusive because they are shared by multiple pathways, e.g., cytokines, or to the extent of cell-cell communication type, paracrine or juxtacrine cell signaling. Likewise, other molecular interactions rather than ligand-receptor mechanisms may need to be set in motion.

LiMd role in transmembrane signaling has been reported (Simons and Toomre, 2000; Suzuki, 2015), with its importance being worth the neologism “glycosynapse” (Regina Todeschini and Hakomori, 2008). Glycosynapse types have been singled out with regard to the interacting components: a LiMd ligand-binding interaction and a direct or reticulated interplay between oligosaccharides belonging to the LiMd cluster and neighboring protean receptor or integrins N-glycan chains (Kopitz, 2017). Altogether, these mechanisms seem to be more related to overall membrane stability, where LiMd could act as a molecular platform which actively organizes either signal molecules or signal interaction assemblies (Brown, 2002; Helms and Zurzolo, 2004; Gupta and Surolia, 2010; Levental and Veatch, 2016).

## 5 Pathological Function of Gb3

### 5.1 Importance of Gb3 as a Transport Toxin Receptor in Microvesicles and Exosomes

It has been established through *in vitro* experiments that the Stx family binds to the Gb3 receptor on the cell membrane surface through its B subunit, causing clathrin-dependent or independent endocytosis which evades the lysosomal degradation pathway. Endosomes containing Stx-Gb3 are retrogradely transported through the Golgi complex to the endoplasmic reticulum, where the Stx subunit cleavage occurs. The A subunit translocates to the cytosol, where it inactivates the ribosome capacity of protein synthesis by its N-glycosidase activity, triggering cell stress and apoptosis (Goldstein et al., 2021).

A novel mechanism through which a cell can evade its toxic content, presumably to prevent a host response, consists in the uptake and kidnapping of Stx by microvesicles through the B subunit that binds to the Gb3 receptor, which requires StxB binding to Gb3 in microvesicles (Willysson et al., 2020). To this end, the toxin translocates from its external location in the microvesicle to its interior. If Stx is localized in the outer membrane, it can be recognized by phagocytes and trigger a host response, which will not occur in the inner area. In HeLa cells, microvesicles have been found to release Stx without ever having passed through the retrograde transport pathway to the ribosomes. Indeed, release may take place even before retrograde transport occurs. It has been observed in patients and mice that cells such as red blood cells can be activated without inducing cytotoxicity or releasing the toxin in microvesicles to be discharged into kidney cells bearing Gb3, thus causing kidney failure (Stahl et al., 2015). Therefore, this mechanism by which the binding or transport of Stx occurs in microvesicles can be protective for donor cells but cytotoxic for recipient ones.

It has been shown that Stxs can be transported in the bloodstream by exosomes–originating from endosomes, as opposed to microvesicles, which originate from Gb3-containing plasma membrane detachments–from D-THP-1, a cell line similar to human macrophages. Accordingly, Stx2 has been found in exosomes from D-THP-1 cells in the endoplasmic reticulum of the human proximal tubule epithelial cell line HK-2 as target cells. Gb3 inhibition in turn reduced exosome uptake into HK-2 cells, which suggests that exosomes derived from D-THP-1 cells are transferred to recipient cells in a Gb3-dependent manner. These Stx2-bearing exosomes caused the phosphorylation of stress-related mitogen-activated protein kinases and cell death by apoptosis, which indicates that the cytotoxicity induced by the exosomes depends on the expression of Gb3 by target cells and also on toxin enzymatic activity. Exosomes were also found to carry pro-inflammatory cytokine mRNAs that could be translated into the recipient cells, which reveals that exosomes heightened Stx2 cytotoxic effects. These data suggest that blocking exosome biogenesis could represent a new therapeutic strategy for the treatment of patients with HUS (Lee et al., 2020).

Given that Stx2-positive microvesicles were reported to be taken up in the murine glomerular endothelium in an EHEC infection model (Stahl et al., 2015), and that mouse glomerular endothelial cells lack Gb3 expression (Psotka et al., 2009), further work provided evidence for microvesicle-mediated Stx2 uptake in cells bearing or lacking endogenous Gb3 (CHO, HeLa or DLD-1). Studies were then conducted to establish whether the presence of Gb3 in microvesicles can single-handedly induce toxin-mediated cellular injury or whether the recipient cell must also express the Gb3 receptor for this to occur. Results show that microvesicles bearing Stx2 lowered metabolism in Gb3-positive cells but not in Gb3-negative ones, which indicates the importance of this receptor in affecting protein synthesis inhibition induced by Gb3-positive Stx2-bearing microvesicles in recipient cells by the same retrograde route as free Stx2 (Johansson et al., 2020). Non-Gb3-expressing cells managed to capture the microvesicles bearing Stx2 but were not susceptible to cytotoxicity. Therefore, endogenous Gb3 seems to be an essential condition for Stx2 contained in microvesicles to be taken up and induce protein inhibition and apoptosis.

### 5.2 Gb3 and Cancer

Human tumor cell types expressing Gb3 include human hematopoietic tumor cell lines, Epstein Barr virus-transformed B cell lines (Furukawa et al., 2002), human cervical cancer (Shin et al., 2009), meningiomas (Salhia et al., 2002) and astrocytoma cell lines (Arab et al., 1998). As these cells have shown significant sensitivity to Stx leading to apoptosis, research has been oriented to employing this toxin as a potential antineoplastic drug. However, a linear correlation between Gb3 expression and Stx cytotoxic sensitivity has not been conclusively established. For instance, astrocytoma SF539 cells show 10 times higher levels of Gb3 expression than U251MG cells but equivalent sensitivity to Stx, while XF498 cells are significantly less sensitive to Stx than U251MG cells while showing 7 times higher Gb3 expression (Arab et al., 1998). This finding could be explained by the fact that, although Gb3 is important for Stx cytotoxic effects, the state of the cell cycle phase seems to be as relevant as absolute Gb3 expression. While total content of Gb3 in Vero cells does not change over the course of the cell cycle, cell surface exposure to Gb3 peaks between the G1 and S phases. This is why Vero cells are 30 times more resistant to Stx in the G0 stationary phase than in cell growth ones, as G1/S is the phase of maximal sensitivity to Stx (Pudymaitis and Lingwood, 1992). This effect may be due to glycolipid membrane distribution and, consequently, the ability to interact with specific molecules (Grant, 1984). In summary, the cryptic nature of Gb3 must be taken into account as a determinant of sensitivity to Stx (Wiels et al., 1984).

The effectiveness of Stx has been assessed following intratumor injection in different tumor xenograft mouse models, which yielded total tumor regression with no apparent adverse effects (Arab et al., 1999; Salhia et al., 2002; Ishitoya et al., 2004; Johansson et al., 2006). In the renal carcinoma cell line AHN, complete tumor regression up to 200 mm^3^ size was observed within 7 days of treatment, and no tumor regrowth was observed after 1 month (Ishitoya et al., 2004). The mechanism underlying tumor regression was the induction of apoptosis, as observed in mouse astrocytoma and stromal blood vessel xenografts (Arab et al., 1999). It is unclear whether blood vessel apoptosis was induced directly by Stx binding to Gb3, by an antiangiogenic effect, by elements released by apoptotic astrocytoma cells or a combination of these mechanisms. Nevertheless, Stx was detected in blood vessels from astrocytoma and other tumors (Arab et al., 1997; Arab et al., 1999), which means that blood vessels from tumor stroma may express Gb3 even if Gb3 expression has not been reported yet (Arab et al., 1999).

Although still elusive in stromal blood vessels of gliomas, Gb3 is certainly expressed in the endothelium and in the media tunica of small arteries and arterioles from tumor cells of Gb3-positive primary human breast cancer biopsies. However, no apparent associations have been found between cell Gb3 expression and TNM classification of malignant tumors, histological type, or hormone receptor expression in human breast cancer (Johansson et al., 2009; Stimmer et al., 2014). Stx has been observed to induce the activation of caspase-9 and caspase-3 apoptotic pathways in glioma and the T47D breast cancer cell line expressing high levels of Gb3. In addition, cell death is blocked by GCS inhibitor PPMP and the pan-caspase inhibitor Z-VAD-fmk, which suggests that Gb3 and caspase-9 and -3 activation are pivotal for cell death. Moreover, Stx induces JNK phosphorylation, a key component of the mitogen-activated protein kinase signaling pathway that controls BAX and mitochondrial function, which may lead to mitochondrial depolarization and caspase-9 activation (Johansson et al., 2006; Johansson et al., 2009).

Gb3 expression has been detected in surgically resected tissue samples of human gastric carcinoma (both intestinal and diffuse types), as well as in endothelial cells from blood vessels and many other gastric cancer cell lines (Geyer et al., 2016; Xu et al., 2019). Recombinant Stx1 plasmid vectors have been used to treat gastric cancer *in vitro* and in nude mouse xenografts, an approach which reduces cancer cell proliferation and tumor growth (Xu et al., 2019).

As an alternative to its use as a Stx target to eliminate tumors, Gb3 has also been proposed as a possible biomarker of tumor invasiveness and/or malignancy. However, Gb3 expression in preinvasive stages as well as invasive tumor samples–as found in testicular tumor samples (Kang et al., 1995), primary ovarian tumors and metastases (Arab et al., 1997), and benignant and malignant meningiomas (Salhia et al., 2002)—makes it an implausible biomarker of tumor invasiveness and/or malignancy. Nevertheless, in other types of cancer, Gb3 may reflect tumor growth and chemotherapy resistance, a predictor of poor prognosis, as high Gb3 expression has been reported in poorly differentiated and chemo-refractory tumors (Arab et al., 1997).

Metastasis is one of the fundamental events in cancer and a major challenge in basic and clinical research. One of the main changes observed in most types of human cancers during disease progression into a metastatic stage is aberrant glycosylation in the cell membrane lipid bilayer of glycosphingolipid composition (Ridley, 2000). Gb3 seems to be involved in cell migration, as human intestinal epithelial Caco-2 and HT29 cells derived from metastatic colon cancer, which express Gb3, present a migratory phenotype with filopodia formation. Furthermore, elimination of Gb3 from colon cancer cells by Gb3-synthase siRNA knockdown completely inhibits cell migration, which shows that Gb3 expression is necessary and sufficient for invasiveness in cell culture models (Kovbasnjuk et al., 2005).

A direct relationship between the expression levels of Gb3 and metastatic capacity has been observed in the T3 murine fibrosarcoma cell line. Gb3 expression level was 10 times higher on the cell surface of highly metastatic T3 cell clones than in the weakly metastatic ones, which suggests that Gb3 may be relevant to the metastatic process (Mannori et al., 1990). Similar findings have been obtained in human colon cancer (Kovbasnjuk et al., 2005).

Some interpretations of the role of Gb3 in tumor cells hint at an evolutionary mechanism of protection against the immune system through which GSLs interfere with leucocyte cytotoxicity (Zhang et al., 2019). For instance, high expression of GSLs by tumor cells results in high levels of GSLs in the tumor microenvironment and in plasma (Ladisch and Wu, 1985; Floutsis et al., 1989). These GSLs may be incorporated by leukocyte membranes, which increases T cell apoptosis rates (Biswas et al., 2009), reduces T cell and NK cell cytotoxicity (Lee et al., 2012; Wegner et al., 2018a), reduces T cell and dendritic cell NF-kappaB signaling (Uzzo et al., 1999), inhibits MHC-II antigen presentation by monocytes (Heitger and Ladisch, 1996), inhibits IL-1B production and the expression of Ig Fc receptor on monocytes and macrophages (Hoon et al., 1989), and promotes a shift in CD4+ T cells from Th1 to a Th2 activated phenotype (Crespo et al., 2006). Conversely, in the presence of free GSLs, microglial TLR4 is downregulated while TLR2 is upregulated, which contributes to inflammatory conditions in the brain (Yoon et al., 2008).

Another protein which seems to be influenced by Gb3 in human tumors is the multidrug resistance (MDR) gene (MDR1) encoding a 170 kDa ATP-dependent drug efflux pump, termed P-glycoprotein (P-gp) (Lucci et al., 1998; De Rosa et al., 2008; Behnam-Motlagh et al., 2010; Johansson et al., 2010; Liu et al., 2010; Tyler et al., 2015; Roy et al., 2020). This pump reduces the intracellular concentration of certain drugs by actively increasing cellular drug efflux (Gottesman and Pastan, 1993; Amawi et al., 2019). Moreover, inhibitors of GSL biosynthesis have been shown to reverse the MDR phenotype (Lavie et al., 1996).

Precursors of Gb3-like glucosylceramide have been proposed as markers of MDR tumors, as they are thought to be required for the acquisition and/or maintenance of MDR (Lavie et al., 1996). GCS inhibitors produce cell death in MDR human KB carcinoma cell lines but not in drug-sensitive KB cell lines, which suggests that glucosylceramide is also essential for MDR KB cell type viability (Nicholson et al., 1999).

In turn, a direct relationship has been reported between MDR1 and Gb3 regulation. Retroviral infection of the MDCK renal cell line with human MDR1 cDNA, encoding the P-gp MDR efflux pump, produces major accumulation of Gb3, sensitivity to Stx and resistance to vinblastine (an antimitotic drug used to treat many kinds of cancer), while P-gp inhibitors prevent Gb3 increase and Stx sensitivity, and concomitantly increase vinblastine sensitivity. However, Gb3 synthase expression is present in both MDR1-MDCK and MDCK-wt and unaffected by P-gp inhibitors, which suggests that increased Gb3 in the cell membrane without a concomitant increase in Gb3 synthase may be explained as a post-translational mechanism. Briefly, GlcCer translocates from the cytosolic face of the Golgi to its lumen face, probably triggered by P-gp. Then, this available GlcCer is the substrate to yield luminal LacCer synthesis and subsequent Gb3 synthesis (Lala et al., 2000); the first glycosylation step in GSL biosynthesis occurs on the cytosolic face of the Golgi apparatus (GCS), and the second step takes place within the lumen of the Golgi (β4Gal-T5), whose product, LacCer, is the precursor for Gb3 and most GSLs (Lannert et al., 1998).

GCS is also overexpressed in breast cancer cells and, as observed in MCF-7 cells, its expression has a direct relationship with P-gp expression and Gb3 accumulation derived from the altered composition of GCS-enriched microdomains. The cellular mechanism involved seems to be mediated by Akt and ERK1/2 signaling, which results in MDR1 upregulation, anti-apoptotic gene activation, and a decrease in pro-apoptotic gene expression. Other pathways reported to regulate MDR1 expression include PKC and PI3K, as inhibition of these two second messengers produce a reduction in MDR1 expression (Wegner et al., 2018a; Wegner et al., 2018b). However, the relationship between Gb3 overexpression and MDR1 expression is much more complex and still to be elucidated.

## 6 Final Considerations

### 6.1 Phylogenetic View

Glycolipids are integral components of the extracellular plane of the membrane lipid bilayer, endomembrane systems such as nucleus and mitochondria (Kozireski-Chuback et al., 1999; Morales et al., 2004; Ledeen and Wu, 2011), and thylakoid/chloroplastic membranes (Janero and Barrnett, 1981; Block et al., 1983; Gombos et al., 1996; Moreau et al., 1998; Boudiere et al., 2014; Petroutsos et al., 2014). Ranging from eubacteria and Archaea to eukaryotic cells, these molecular arrangements constitute a biochemical platform for varied and crucial functions in physiology regarding cellular communication, adhesion, growth, and motility; functional receptors, second messengers, and signaling modulators; and apoptosis and cell cycle regulators or gauges in harsh environments (Schnaar et al., 2009; Lombard et al., 2012; Guimaraes et al., 2014; Hori et al., 2016). Glycan diversity in nature has been neglected to a certain extent, and glycolipid evolution is a complex process featuring both commonality between distantly related taxa and structural diversity within and between evolutionary lineages. While many taxa have no information on their glycolipid profiles, others might be wholly characterized by a particular glycolipid pattern and simultaneously display biochemical variations at a subdivision level. For instance, the glycolipid structure exhibited by Eubacteria and Archaea have relatively little in common with that of eukaryotes and, at the same time, glycolipid types identified in mammalian cells have forms related with other eukaryotes and Archaea (Varki et al., 2009; Hori et al., 2016). Glycolipids include diacylglycerol- or alkylacylglycerol-linked glycoglycerolipids, ceramide-linked GSLs, and phosphatidylinositol-linked glycosylphosphatidylinositols (GPIs). The first group represents the most abundant type in Eubacteria, Archaea and Plantae taxa (Holzl and Dormann, 2007; Gabius, 2011), while GSLs and GPIs appear in Animalia, Protista, Fungi and Plantae taxa (Varki et al., 2009; Guimaraes et al., 2014). In sum, glycolipids’ intrinsic structural features, the strong relationship they bear with their enzymatic synthesis/degradation pathways and glycolipid assembly and disassembly gene sequencing could lead to comprehensive and fruitful phylogenetic analysis (Petit et al., 2021).

### 6.2 Physiological Wrap Up

So far, the molecular structure has spotlighted GSLs physiological function, as their motif diversity and allocation are plausible arguments for involving GSLs in intercellular interaction, cell adhesion, and intracellular signaling. In addition, GSLs act as functional and topological platforms, glycosynapses and LiMd, respectively, allowing particular interactions with receptors or other glycans and membrane spatial organization (Kopitz et al., 2014; Ruiz et al., 2014; Ludwig et al., 2016). In contrast with comprehensive studies on gangliosides, Gb have been largely restricted to their role as toxin-binding sites and deserve more thorough research in pursuit of in-depth knowledge.

A functional message coded beneath GSL structure prompted glycoscience research to elaborate on the ubiquitous and fundamental biological roles of glycans (Gabius et al., 2015; Solis et al., 2015). The use of antibodies against the whole cell or purified compounds are experimental confirmation to prove glycans as clusters of differentiation markers. Glycans molecular recognition properties include binding mechanisms as well as conformational ones (Shimizu, 2003). In this context, membrane modeling was developed to study GSL-receptor function and lipid structure/microenvironment inter-reliance, such as cholesterol-enriched microdomains (Mahfoud et al., 2010), and showed that cell membrane remodeling processes and GSL-receptor function establish a bidirectional dialog. Accordingly, GSL fatty acid heterogeneity may participate in LiMd organization and thus modify GSL-receptor biological behavior (Lingwood et al., 2010b). In this scenario, GSL pattern distribution might act as a physiological *in vivo* flag as well as a pathological one (Lingwood et al., 2010a).

Further GSL functional significance has been obtained using chemically programmable glycodendrisomes, a self-assembled structure similar to a cell membrane, where different compounds, in nature and density, actively engage in cellular response (Percec et al., 2013; Zhang et al., 2015a; Zhang et al., 2015b; Xiao et al., 2016). These data describe GSL distribution not only as a potential flag but also as a biochemical tuner for the biological functions mentioned above (see Section 6.1).

### 6.3 Efficacy of Gb3 Targeting to Neutralize Stx Cytotoxic Action and Neurodegenerative Diseases

A knock-out mouse design for the Gb3 synthase gene involved in the synthesis of the Gb3 receptor (Figure 1) has shown that this receptor mediates tissue damage and the pathological characteristics produced by Stx. As null mutant mice showed no morphological or functional changes, the structures of the remaining glycolipids may be thought to compensate for the glycolipid functions of the globo-series. Therefore, blocking the function of Gb3 has been proposed as an effective method to interrupt the deleterious effects of EHEC poisoning (Okuda et al., 2006).

As a means of neutralizing the deleterious effects of Stx, several strategies have been developed to block the Gb3 receptor and prevent its binding. While most approaches have been successful in *in vitro* and animal models, none of the developments have shown therapeutic efficacy.

One of the first therapies to neutralize the effect of Stx was the blocking the Gb3 receptor from the intestinal lumen. To this end, a drug was designed comprising the trisaccharide part of Gb3 (the portion of the molecule that binds to Stx) bound to silica dioxide particles, which was called Synsorb ^®^ Pk (Synsorb ^®^ Biotech). This drug failed in pediatric patients, as it was unable to neutralize all the Stx molecules. Therefore, traces of Stx still produced diarrheagenic HUS, which may need to be addressed through the use of Gb3 analogs and anti-Stx antibodies in the bloodstream (Armstrong et al., 1991; Trachtman et al., 2003).

In addition, a pentameric mold called “starfish” was designed with 2 B subunits in which disaccharides galactose α-1,4 galactose are joined in the form of a sandwich. Starfish prevented Stx binding to Gb3 *in vitro* and in animal models. Also, Gb3 multi-oligosaccharides mounted on synthetic materials proved effective *in vitro* and in mice, but acrylamide toxicity made them clinically unfeasible. Peptide libraries were designed to mimic Gb3 for *in vitro* studies, and liposomes containing Gb3 sugars coupled to phosphatidylethanolamine were effective in neutralizing Stx cytotoxicity *in vitro*. Moreover, constructs of bacterial lipooligosaccharides mimicked the sugar sequence of Gb3 and were found to be protective *in vitro* and in animal models. Nevertheless, despite their effectiveness in experimental models, these devices have never been clinically tested (Lingwood, 2020).

Water-soluble Gb3 analogs have also been designed replacing the fatty acid portion with an adamantane frame to target one of the B subunit binding sites. These modifications retained the biological activity of the GSL in the membrane and succeeded in preventing GSL biosynthesis. These analogs also bound the B subunit of Stx2 more effectively than Gb3 and blocked Stx-Gb3 binding *in vitro*. However, this protective effect was not observed in a mouse experimental model (for more detailed information see Lingwood 2020) (Lingwood, 2020).

Therefore, there has been no success to date in developing an effective analog of Gb3 to neutralize the deleterious effects of Stx by blocking its Gb3 receptor for clinical purposes. At present, expectation has been placed on the design of antibodies as the most promising strategy.

Gb3 has been recently reported as a novel target to prevent neuronal secretion of amyloid beta (Aß), a key factor of Alzheimer’s disease. The amyloid precursor protein (APP) is known to be located in microdomains of lipid rafts, much like the Gb3 receptor. APP is internalized into early endosomes and then into late/recycling endosomes, where it is cleaved by secretases to yield Aß, which is released into the extracellular space in a free or exosome-mediated manner. Strikingly, Gb3 is internalized in the same early endosomes as APP, and they share common features of intracellular transport. A mutant nontoxigenic Stx2 (mStx2) lacking cytotoxic activity but still binding to Gb3 has been designed for incubation in CHO cells transiently expressing APP. As reported, mStx2a incubation formed clusters of Gb3 promoting the assembly of APP into lipid rafts of the same endosomes. mStx2a was observed to facilitate the transfer of APP to the lysosomal acidic compartment, reducing the amount of APP and inhibiting the production and extracellular release of Aß. Even though the precise mechanisms underlying this process still need to be determined, the prevention of Aß release might be a novel strategy for protection against Alzheimer’s disease through the Gb3 receptor (Sato et al., 2021).

## 7 Conclusion and Perspectives

The present work provides the latest findings on the role of Gb3 in particular, and GSLs in general, in cell signaling, adhesion and communication, as well as their participation in a wide range of immune and metabolic processes. To understand its role in cell functionality, it is necessary to elucidate how Gb3 is formed and degraded, what happens when these processes fail, and the inflammatory framework involved.

This review also presents an overall description of the cell types expressing Gb3 and a detailed account of its physiological function in immune and renal cells. An update of the pathological mechanisms involving Gb3 is also provided, as findings of Gb3 in exosomes following intoxication with Stx have paved the way for new therapeutic challenges and perspectives. In addition, the design of antibodies to block the binding of Stx to its receptor Gb3 has recently sparked interest as the most promising strategy against HUS, while the state of the cell cycle phase has gained relevance comparable to absolute Gb3 expression for cancer treatment in tumor cells bearing Gb3.

In this way, the present review broadens the understanding of Gb3 function beyond its role in cell membrane structure in lipid raft microdomains, infectious and neurodegenerative diseases, and cancer, and unveils new features for Gb3, as part of the GSL group, from a biological, physiological and immunity point of view with potential for basic research and translation into clinical applications. This review also calls for attention to expand the paradigm based on models of nucleic acid and protein functionality to those including saccharides and lipids, to find hidden solutions to traditional unsolved pathologies. Novel knowledge of these molecules will impact the understanding of cellular functionality and generate new perspectives of their role in metabolism, development, differentiation, and evolution.

## References

[B1] Abe A. Gregory S. Lee L. Killen P. D. Brady R. O. Kulkarni A. (2000). Reduction of Globotriaosylceramide in Fabry Disease Mice by Substrate Deprivation. J. Clin. Invest. 105 (11), 1563–1571. 10.1172/JCI9711 10841515 PMC300859

[B2] Allende M. L. Proia R. L. (2014). Simplifying Complexity: Genetically Resculpting Glycosphingolipid Synthesis Pathways in Mice to Reveal Function. Glycoconj J. 31 (9), 613–622. 10.1007/s10719-014-9563-5 25351657 PMC4245496

[B3] Amawi H. Sim H.-M. Tiwari A. K. Ambudkar S. V. Shukla S. (2019). ABC Transporter-Mediated Multidrug-Resistant Cancer. Adv. Exp. Med. Biol. 1141, 549–580. 10.1007/978-981-13-7647-4_12 31571174

[B4] Arab S. Russel E. Chapman W. B. Rosen B. Lingwood C. A. (1997). Expression of the Verotoxin Receptor Glycolipid, Globotriaosylceramide, in Ovarian Hyperplasias. Oncol. Res. 9 (10), 553–563. 9507533

[B5] Arab S. Rutka J. Lingwood C. (1999). Verotoxin Induces Apoptosis and the Complete, Rapid, Long-Term Elimination of Human Astrocytoma Xenografts in Nude Mice. Oncol. Res. 11 (1), 33–39. 10451029

[B6] Arab S. Lingwood C. A. (1998). Intracellular Targeting of the Endoplasmic Reticulum/nuclear Envelope by Retrograde Transport May Determine Cell Hypersensitivity to Verotoxin via Globotriaosyl Ceramide Fatty Acid Isoform Traffic. J. Cel. Phys.CO 177 (4), 646–660. 10.1002/(SICI)1097-4652 10092217

[B7] Arab S. Murakami M. Dirks P. Boyd B. Hubbard S. L. Lingwood C. A. (1998). Verotoxins Inhibit the Growth of and Induce Apoptosis in Human Astrocytoma Cells. J. Neurooncol. 40 (2), 137–150. 10.1023/a:1006010019064 9892096

[B8] Armstrong G. D. Fodor E. Vanmaele R. (1991). Investigation of Shiga-like Toxin Binding to Chemically Synthesized Oligosaccharide Sequences. J. Infect. Dis. 164 (6), 1160–1167. 10.1093/infdis/164.6.1160 1659599

[B9] Author anonymous (1978). The Nomenclature of Lipids (Recommendations 1976) IUPAC-IUB Commission on Biochemical Nomenclature. J. Lipid. Res. 19 (1), 114–128. https://pubmed.ncbi.nlm.nih.gov/621435/ 621435

[B10] Behnam-Motlagh P. Tyler A. Grankvist K. Johansson A. (2010). Verotoxin-1 Treatment or Manipulation of its Receptor Globotriaosylceramide (Gb3) for Reversal of Multidrug Resistance to Cancer Chemotherapy. Toxins 2 (10), 2467–2477. 10.3390/toxins2102467 22069561 PMC3153170

[B11] Berdasco C. Duhalde Vega M. Rosato-Siri M. V. Goldstein J. (2019). Environmental Cues Modulate Microglial Cell Behavior Upon Shiga Toxin 2 From Enterohemorrhagic *Escherichia coli* Exposure. Front. Cell Infect. Microbiol. 9, 442. 10.3389/fcimb.2019.00442 31970091 PMC6960108

[B12] Binnington B. Lingwood D. Nutikka A. Lingwood C. A. (2002). Effect of Globotriaosyl Ceramide Fatty Acid Alpha-Hydroxylation on the Binding by Verotoxin 1 and Verotoxin 2. Neurochem. Res. 27 (7-8), 807–813. 10.1023/a:1020261125008 12374217

[B13] Binz T. Rummel A. (2009). Cell Entry Strategy of Clostridial Neurotoxins. J. Neurochem. 109 (6), 1584–1595. 10.1111/j.1471-4159.2009.06093.x 19457120

[B14] Biswas S. Biswas K. Richmond A. Ko J. Ghosh S. Simmons M. (2009). Elevated Levels of Select Gangliosides in T Cells from Renal Cell Carcinoma Patients Is Associated with T Cell Dysfunction. J. Immunol. 183 (8), 5050–5058. 10.4049/jimmunol.0900259 19801523 PMC2890308

[B15] Block M. A. Dorne A. J. Joyard J. Douce R. (1983). Preparation and Characterization of Membrane Fractions Enriched in Outer and Inner Envelope Membranes from Spinach Chloroplasts. II. Biochemical Characterization. J. Biol. Chem. 258 (21), 13281–13286. 10.1016/s0021-9258(17)44113-5 6630230

[B16] Boudière L. Michaud M. Petroutsos D. Rébeillé F. Falconet D. Bastien O. (2014). Glycerolipids in Photosynthesis: Composition, Synthesis and Trafficking. Biochim. Biophys. Acta (Bba) - Bioenerg. 1837 (4), 470–480. 10.1016/j.bbabio.2013.09.007 24051056

[B17] Breiden B. Sandhoff K. (2019). Lysosomal Glycosphingolipid Storage Diseases. Annu. Rev. Biochem. 88, 461–485. 10.1146/annurev-biochem-013118-111518 31220974

[B18] Brown D. (2001). Structure and Function of Membrane Rafts. Int. J. Med. Microbiol. 291 (6-7), 433–437. 10.1078/1438-4221-00150 11890541

[B19] Budani M. Auray-Blais C. Lingwood C. (2021). ATP-binding Cassette Transporters Mediate Differential Biosynthesis of Glycosphingolipid Species. J. Lipid Res. 62, 100128. 10.1016/j.jlr.2021.100128 34597626 PMC8569594

[B20] Chakrabandhu K. Huault S. Garmy N. Fantini J. Stebe E. Mailfert S. (2008). The Extracellular Glycosphingolipid-Binding Motif of Fas Defines its Internalization Route, Mode and Outcome of Signals upon Activation by Ligand. Cell Death Differ 15 (12), 1824–1837. 10.1038/cdd.2008.115 18670435

[B21] Chan B. Adam D. N. (2018). A Review of Fabry Disease. Skin Ther. Lett 23 (2), 4–6. 29562089

[B22] Cohen A. Hannigan G. E. Williams B. R. Lingwood C. A. (1987). Roles of Globotriosyl- and Galabiosylceramide in Verotoxin Binding and High Affinity Interferon Receptor. J. Biol. Chem. 262 (35), 17088–17091. 10.1016/s0021-9258(18)45495-6 2824515

[B23] Cooling L. L. W. Walker K. E. Gille T. Koerner T. A. W. (1998). Shiga Toxin Binds Human Platelets via Globotriaosylceramide (P K Antigen) and a Novel Platelet Glycosphingolipid. Infect. Immun. 66 (9), 4355–4366. 10.1128/IAI.66.9.4355-4366.1998 9712788 PMC108526

[B24] Crespo F. A. Sun X. Cripps J. G. Fernandez-Botran R. (2006). The Immunoregulatory Effects of Gangliosides Involve Immune Deviation Favoring Type-2 T Cell Responses. J. Leukoc. Biol. 79 (3), 586–595. 10.1189/jlb.0705395 16415169

[B25] D'Angelo G. Capasso S. Sticco L. Russo D. (2013). Glycosphingolipids: Synthesis and Functions. FEBS J. 280 (24), 6338–6353. 10.1111/febs.12559 24165035

[B26] De Rosa M. F. Ackerley C. Wang B. Ito S. Clarke D. M. Lingwood C. (2008). Inhibition of Multidrug Resistance by Adamantylgb3, a Globotriaosylceramide Analog. J. Biol. Chem. 283 (8), 4501–4511. 10.1074/jbc.M705473200 18003606

[B27] Del Nagro C. J. Otero D. C. Anzelon A. N. Omori S. A. Kolla R. V. Rickert R. C. (2005). CD19 Function in central and Peripheral B-Cell Development. Ir 31 (2), 119–132. 10.1385/IR:31:2:119 15778510

[B28] Đevenica D. Čikeš Čulić V. Vuica A. Markotić A. (2011). Biochemical, Pathological and Oncological Relevance of Gb3Cer Receptor. Med. Oncol. 28 (Suppl. 1), 675–684. 10.1007/s12032-010-9732-8 21069478

[B29] Ergonul Z. Hughes A. K. Kohan D. E. (2003). Induction of Apoptosis of Human Brain Microvascular Endothelial Cells by Shiga Toxin 1. J. Infect. Dis. 187 (1), 154–158. 10.1086/345861 12508161

[B30] Erwert R. D. Eiting K. T. Tupper J. C. Winn R. K. Harlan J. M. Bannerman D. D. (2003). Shiga Toxin Induces Decreased Expression of the Anti-apoptotic Protein Mcl-1 Concomitant with the Onset of Endothelial Apoptosis. Microb. Pathogenesis 35 (2), 87–93. 10.1016/s0882-4010(03)00100-1 12901848

[B31] Ewers H. Helenius A. (2011). Lipid-mediated Endocytosis. Cold Spring Harbor Perspect. Biol. 3 (8)–a004721. 10.1101/cshperspect.a004721 PMC3140687 21576253

[B32] Fantini J. Maresca M. Hammache D. Yahi N. Delezay O. (2000). Glycosphingolipid (GSL) Microdomains as Attachment Platforms for Host Pathogens and Their Toxins on Intestinal Epithelial Cells: Activation of Signal Transduction Pathways and Perturbations of Intestinal Absorption and Secretion. Glycoconj J. 17 (3 -4), 173–179. 10.1023/a:1026580905156 11201788

[B33] Fas S. C. Fritzsching B. Suri-Payer E. Krammer P. H. (2005). Death Receptor Signaling and its Function in the Immune System. Curr. Dir. Autoimmun. 9, 1–17. 10.1159/000090767 16394652

[B34] Floutsis G. Ulsh L. Ladisch S. (1989). Immunosuppressive Activity of Human Neuroblastoma Tumor Gangliosides. Int. J. Cancer 43 (1), 6–9. 10.1002/ijc.2910430103 2910832

[B35] Fraser M. E. Fujinaga M. Cherney M. M. Melton-Celsa A. R. Twiddy E. M. O'Brien A. D. (2004). Structure of Shiga Toxin Type 2 (Stx2) from *Escherichia coli* O157:H7. J. Biol. Chem. 279 (26), 27511–27517. 10.1074/jbc.M401939200 15075327

[B36] Fujii J. Kinoshita Y. Kita T. Higure A. Takeda T. Tanaka N. (1996). Magnetic Resonance Imaging and Histopathological Study of Brain Lesions in Rabbits Given Intravenous Verotoxin 2. Infect. Immun. 64 (12), 5053–5060. 10.1128/iai.64.12.5053-5060.1996 8945546 PMC174488

[B37] Fujii J. Kita T. Yoshida S. Takeda T. Kobayashi H. Tanaka N. (1994). Direct Evidence of Neuron Impairment by Oral Infection with Verotoxin-Producing *Escherichia coli* O157:H- in Mitomycin-Treated Mice. Infect. Immun. 62 (8), 3447–3453. 10.1128/iai.62.8.3447-3453.1994 8039916 PMC302977

[B38] Fujii J. Matsui T. Heatherly D. P. Schlegel K. H. Lobo P. I. Yutsudo T. (2003). Rapid Apoptosis Induced by Shiga Toxin in HeLa Cells. Infect. Immun. 71 (5), 2724–2735. 10.1128/IAI.71.5.2724-2735.2003 12704147 PMC153243

[B39] Furukawa K. Yokoyama K. Sato T. Wiels J. Hirayama Y. Ohta M. (2002). Expression of the Gb3/CD77 Synthase Gene in Megakaryoblastic Leukemia Cells. J. Biol. Chem. 277 (13), 11247–11254. 10.1074/jbc.M109519200 11782470

[B40] Gabius H.-J. Kaltner H. Kopitz J. André S. (2015). The Glycobiology of the CD System: a Dictionary for Translating Marker Designations into Glycan/lectin Structure and Function. Trends Biochem. Sci. 40 (7), 360–376. 10.1016/j.tibs.2015.03.013 25981696

[B41] Gabius H.-J. (2011). The Sugar Code Fundamentals of Glycosciences. Weinheim: Wiley.

[B42] García A. Bosques C. J. Wishnok J. S. Feng Y. Karalius B. J. Butterton J. R. (2006). Renal Injury Is a Consistent Finding in Dutch Belted Rabbits Experimentally Infected with EnterohemorrhagicEscherichia Coli. J. Infect. Dis. 193 (8), 1125–1134. 10.1086/501364 16544253

[B43] George T. Boyd B. Price M. Lingwood C. Maloney M. (2001). MHC Class II Proteins Contain a Potential Binding Site for the Verotoxin Receptor Glycolipid CD77. Cel Mol Biol (Noisy-le-grand) 47 (7), 1179–1185. 11838965

[B44] Geyer P. E. Maak M. Nitsche U. Perl M. Novotny A. Slotta-Huspenina J. (2016). Gastric Adenocarcinomas Express the Glycosphingolipid Gb3/CD77: Targeting of Gastric Cancer Cells with Shiga Toxin B-Subunit. Mol. Cancer Ther. 15 (5), 1008–1017. 10.1158/1535-7163.MCT-15-0633 26826119

[B45] Goldstein J. Nuñez-Goluboay K. Pinto A. (2020). Therapeutic Strategies to Protect the Central Nervous System against Shiga Toxin from Enterohemorrhagic *Escherichia coli* . Cn 19 (1), 24–44. 10.2174/1570159X18666200220143001 PMC7903495 32077828

[B46] Gombos Z. Wada H. Varkonyi Z. Los D. A. Murata N. (1996). Characterization of the Fad12 Mutant of Synechocystis that Is Defective in Δ12 Acyl-Lipid Desaturase Activity. Biochim. Biophys. Acta (Bba) - Lipids Lipid Metab. 1299 (1), 117–123. 10.1016/0005-2760(95)00204-9 8555244

[B47] Gottesman M. M. Pastan I. (1993). Biochemistry of Multidrug Resistance Mediated by the Multidrug Transporter. Annu. Rev. Biochem. 62, 385–427. 10.1146/annurev.bi.62.070193.002125 8102521

[B48] Grant C. W. M. (1984). Cell Surface Structural Implications of Some Experiments with Isolated Glycolipids and Glycoproteins. Can. J. Biochem. Cel Biol. 62 (11), 1151–1157. 10.1139/o84-148 6098359

[B49] GuimarÃ£es L. L. Toledo M. S. Ferreira F. A. S. Straus A. H. Takahashi H. K. (2014). Structural Diversity and Biological Significance of Glycosphingolipids in Pathogenic and Opportunistic Fungi. Front. Cel. Infect. Microbiol. 4, 138. 10.3389/fcimb.2014.00138 PMC4174763 25309884

[B50] Gupta G. Surolia A. (2010). Glycosphingolipids in Microdomain Formation and Their Spatial Organization. FEBS Lett. 584 (9), 1634–1641. 10.1016/j.febslet.2009.11.070 19941856

[B51] Hakomori S. (2003). Structure, Organization, and Function of Glycosphingolipids in Membrane. Curr. Opin. Hematol. 10 (1), 16–24. 10.1097/00062752-200301000-00004 12483107

[B52] Hammache D. Yahi N. Maresca M. Piéroni G. Fantini J. (1999). Human Erythrocyte Glycosphingolipids as Alternative Cofactors for Human Immunodeficiency Virus Type 1 (HIV-1) Entry: Evidence for CD4-Induced Interactions between HIV-1 Gp120 and Reconstituted Membrane Microdomains of Glycosphingolipids (Gb3 and GM3). J. Virol. 73 (6), 5244–5248. 10.1128/JVI.73.6.5244-5248.1999 10233996 PMC112578

[B53] Hannigan G. E. Gewert D. R. Williams B. R. (1984). Characterization and Regulation of Alpha-Interferon Receptor Expression in Interferon-Sensitive and -resistant Human Lymphoblastoid Cells. J. Biol. Chem. 259 (15), 9456–9460. 10.1016/s0021-9258(17)42722-0 6086635

[B54] Heitger A. Ladisch S. (1996). Gangliosides Block Antigen Presentation by Human Monocytes. Biochim. Biophys. Acta (Bba) - Lipids Lipid Metab. 1303 (2), 161–168. 10.1016/0005-2760(96)00091-4 8856046

[B55] Helms J. B. Zurzolo C. (2004). Lipids as Targeting Signals: Lipid Rafts and Intracellular Trafficking. Traffic 5 (4), 247–254. 10.1111/j.1600-0854.2004.0181.x 15030566

[B56] Herzer S. Meldner S. Rehder K. Gröne H.-J. Nordström V. (2016). Lipid Microdomain Modification Sustains Neuronal Viability in Models of Alzheimer's Disease. Acta Neuropathol. Commun. 4 (1), 103. 10.1186/s40478-016-0354-z 27639375 PMC5027102

[B57] Högerkorp C.-M. Borrebaeck C. A. K. (2006). The Human CD77− B Cell Population Represents a Heterogeneous Subset of Cells Comprising Centroblasts, Centrocytes, and Plasmablasts, Prompting Phenotypical Revision. J. Immunol. 177 (7), 4341–4349. 10.4049/jimmunol.177.7.4341 16982868

[B58] Hölzl G. Dörmann P. (2007). Structure and Function of Glycoglycerolipids in Plants and Bacteria. Prog. Lipid Res. 46 (5), 225–243. 10.1016/j.plipres.2007.05.001 17599463

[B59] Honigmann A. Pralle A. (2016). Compartmentalization of the Cell Membrane. J. Mol. Biol. 428 (24 Pt A), 4739–4748. 10.1016/j.jmb.2016.09.022 27720722

[B60] Hoon D. S. B. Jung T. Naungayan J. Cochran A. J. Morton D. L. McBride W. H. (1989). Modulation of Human Macrophage Functions by Gangliosides. Immunol. Lett. 20 (4), 269–275. 10.1016/0165-2478(89)90034-5 2785500

[B61] Hori K. Nobusawa T. Watanabe T. Madoka Y. Suzuki H. Shibata D. (2016). Tangled Evolutionary Processes with Commonality and Diversity in Plastidial Glycolipid Synthesis in Photosynthetic Organisms. Biochim. Biophys. Acta (Bba) - Mol. Cel Biol. Lipids 1861 (9 Pt B), 1294–1308. 10.1016/j.bbalip.2016.04.015 27108062

[B62] Ishitoya S. Kurazono H. Nishiyama H. Nakamura E. Kamoto T. Habuchi T. (2004). Verotoxin Induces Rapid Elimination of Human Renal Tumor Xenografts in SCID Mice. J. Urol. 171 (3), 1309–1313. 10.1097/01.ju.0000100110.11129.85 14767339

[B63] Ivashkiv L. B. Donlin L. T. (2014). Regulation of Type I Interferon Responses. Nat. Rev. Immunol. 14 (1), 36–49. 10.1038/nri3581 24362405 PMC4084561

[B64] Iwamura K. Furukawa K. Uchikawa M. Sojka B. N. Kojima Y. Wiels J. (2003). The Blood Group P1 Synthase Gene Is Identical to the Gb3/CD77 Synthase Gene. J. Biol. Chem. 278 (45), 44429–44438. 10.1074/jbc.M301609200 12888565

[B65] Janero D. R. Barrnett R. (1981). Cellular and Thylakoid-Membrane Phospholipids of Chlamydomonas Reinhardtii 137+. J. Lipid Res. 22 (7), 1126–1130. 10.1016/s0022-2275(20)40671-6 7299293

[B66] Jennemann R. Federico G. Mathow D. Rabionet M. Rampoldi F. Popovic Z. V. (2017). Inhibition of Hepatocellular Carcinoma Growth by Blockade of Glycosphingolipid Synthesis. Oncotarget 8 (65), 109201–109216. 10.18632/oncotarget.22648 29312601 PMC5752514

[B67] Johansson D. Andersson C. Moharer J. Johansson A. Behnam-Motlagh P. (2010). Cisplatin-induced Expression of Gb3 Enables Verotoxin-1 Treatment of Cisplatin Resistance in Malignant Pleural Mesothelioma Cells. Br. J. Cancer 102 (2), 383–391. 10.1038/sj.bjc.6605467 20010943 PMC2816648

[B68] Johansson D. Johansson A. Grankvist K. Andersson U. Henriksson R. Bergström P. (2006). Verotoxin-1 Induction of Apoptosis in Gb3-Expressing Human Glioma Cell Lines. Cancer Biol. Ther. 5 (9), 1211–1217. 10.4161/cbt.5.9.3173 16929170

[B69] Johansson D. Kosovac E. Moharer J. Ljuslinder I. Brännström T. Johansson A. (2009). Expression of Verotoxin-1 Receptor Gb3 in Breast Cancer Tissue and Verotoxin-1 Signal Transduction to Apoptosis. BMC Cancer 9, 67. 10.1186/1471-2407-9-67 19245689 PMC2650710

[B70] Johansson K. Willysson A. Kristoffersson A.-C. Tontanahal A. Gillet D. Ståhl A.-l. (2020). Shiga Toxin-Bearing Microvesicles Exert a Cytotoxic Effect on Recipient Cells Only when the Cells Express the Toxin Receptor. Front. Cel. Infect. Microbiol. 10, 212. 10.3389/fcimb.2020.00212 PMC7261856 32523894

[B71] Jones N. L. Islur A. Haq R. Mascarenhas M. Karmali M. A. Perdue M. H. (2000). Escherichia coliShiga Toxins Induce Apoptosis in Epithelial Cells that Is Regulated by the Bcl-2 Family. Am. J. Physiology-Gastrointestinal Liver Physiol. 278 (5), G811–G819. 10.1152/ajpgi.2000.278.5.G811 10801274

[B72] Kang J. Rajpert-De Meyts E. Skakkeb�k N. Wiels J. (1995). Expression of the Glycolipid Globotriaosylceramide (Gb3) in Testicular Carcinoma *In Situ* . Vichows Archiv A. Pathol. Anat. 426 (4), 369–374. 10.1007/BF00191346 7599789

[B73] Karlsson K.-A. (1989). Animal Glycosphingolipids as Membrane Attachment Sites for Bacteria. Annu. Rev. Biochem. 58, 309–350. 10.1146/annurev.bi.58.070189.001521 2673013

[B74] Karmali M. A. Petric M. Lim C. Fleming P. C. Arbus G. S. Lior H. (1985). The Association between Idiopathic Hemolytic Uremic Syndrome and Infection by Verotoxin-Producing *Escherichia coli* . J. Infect. Dis. 151 (5), 775–782. 10.1093/infdis/151.5.775 3886804

[B75] Karpman D. Håkansson A. Perez M.-T. R. Isaksson C. Carlemalm E. Caprioli A. (1998). Apoptosis of Renal Cortical Cells in the Hemolytic-Uremic Syndrome: *In Vivo* and *In Vitro* Studies. Infect. Immun. 66 (2), 636–644. 10.1128/IAI.66.2.636-644.1998 9453620 PMC107951

[B76] Katz H. R. Schwarting G. A. LeBlanc P. A. Austen K. F. Stevens R. L. (1985). Identification of the Neutral Glycosphingolipids of Murine Mast Cells: Expression of Forssman Glycolipid by the Serosal but Not the Bone Marrow-Derived Subclass. J. Immunol. 134 (4), 2617–2623. 3871816

[B77] Keusch J. J. Manzella S. M. Nyame K. A. Cummings R. D. Baenziger J. U. (2000). Expression Cloning of a New Member of the ABO Blood Group Glycosyltransferases, iGb3 Synthase, that Directs the Synthesis of Isoglobo-Glycosphingolipids. J. Biol. Chem. 275 (33), 25308–25314. 10.1074/jbc.M002629200 10854427

[B78] Khine A.-A. Lingwood C. A. (2000). Functional Significance of Globotriaosyl Ceramide in Interferon-?2/type 1 Interferon Receptor-Mediated Antiviral Activity. J. Cel. Phys.CO 182 (1), 97–108. 10.1002/(SICI)1097-4652 10567921

[B79] Kiguchi K. Henning-Chubb C. B. Huberman E. (1990). Glycosphingolipid Patterns of Peripheral Blood Lymphocytes, Monocytes, and Granulocytes Are Cell Specific1. J. Biochem. 107 (1), 8–14. 10.1093/oxfordjournals.jbchem.a123016 2332423

[B80] Kioka N. Minami K. Tamura A. Yoshikawa N. (2012). Chemokine Expression in Human Astrocytes in Response to Shiga Toxin 2. Int. J. Inflamm. 2012, 1–9. 10.1155/2012/135803 PMC3529876 23304632

[B81] Kjellberg M. A. Mattjus P. (2013). Glycolipid Transfer Protein Expression Is Affected by Glycosphingolipid Synthesis. PLoS One 8 (7), e70283. 10.1371/journal.pone.0070283 23894633 PMC3722133

[B82] Klein U. Tu Y. Stolovitzky G. A. Keller J. L. Haddad J. Jr. Miljkovic V. (2003). Transcriptional Analysis of the B Cell Germinal center Reaction. Proc. Natl. Acad. Sci. 100 (5), 2639–2644. 10.1073/pnas.0437996100 12604779 PMC151393

[B83] Knapp W. Dorken B. Rieber P. Schmidt R. Stein H. von dem Borne A. (1989). CD Antigens 1989. Blood 74 (4), 1448–1450. 10.1182/blood.v74.4.1448.bloodjournal7441448 2765668

[B84] Kniep B. Monner D. A. Schwulera U. Muhlradt P. F. (1985). Glycosphingolipids of the Globo-Series Are Associated with the Monocytic Lineage of Human Myeloid Cells. Eur. J. Biochem. 149 (1), 187–191. 10.1111/j.1432-1033.1985.tb08910.x 3858098

[B85] Kojima Y. Fukumoto S. Furukawa K. Okajima T. Wiels J. Yokoyama K. (2000). Molecular Cloning of globotriaosylceramide/CD77 Synthase, a Glycosyltransferase that Initiates the Synthesis of Globo Series Glycosphingolipids. J. Biol. Chem. 275 (20), 15152–15156. 10.1074/jbc.M909620199 10748143

[B86] Kolling G. L. Obata F. Gross L. K. Obrig T. G. (2008). Immunohistologic Techniques for Detecting the Glycolipid Gb3 in the Mouse Kidney and Nervous System. Histochem. Cel Biol 130 (1), 157–164. 10.1007/s00418-008-0417-8 18365234

[B87] Kopitz J. (2017). Lipid Glycosylation: a Primer for Histochemists and Cell Biologists. Histochem. Cel Biol 147 (2), 175–198. 10.1007/s00418-016-1518-4 27999995

[B88] Kopitz J. Vértesy S. André S. Fiedler S. Schnölzer M. Gabius H.-J. (2014). Human Chimera-type Galectin-3: Defining the Critical Tail Length for High-Affinity Glycoprotein/cell Surface Binding and Functional Competition with Galectin-1 in Neuroblastoma Cell Growth Regulation. Biochimie 104, 90–99. 10.1016/j.biochi.2014.05.010 24909114

[B89] Kovbasnjuk O. Mourtazina R. Baibakov B. Wang T. Elowsky C. Choti M. A. (2005). The Glycosphingolipid Globotriaosylceramide in the Metastatic Transformation of colon Cancer. Proc. Natl. Acad. Sci. 102 (52), 19087–19092. 10.1073/pnas.0506474102 16365318 PMC1323164

[B90] Kozireski-Chuback D. Wu G. Ledeen R. W. (1999). Developmental Appearance of Nuclear GM1 in Neurons of the central and Peripheral Nervous Systems. Develop. Brain Res. 115 (2), 201–208. 10.1016/s0165-3806(99)00062-0 10407137

[B91] Ladisch S. Wu Z.-L. (1985). Detection of a Tumour-Associated Ganglioside in Plasma of Patients with Neuroblastoma. The Lancet 325 (8421), 136–138. 10.1016/s0140-6736(85)91906-3 PMC4186706 2857215

[B92] Lala P. Ito S. Lingwood C. A. (2000). Retroviral Transfection of Madin-Darby Canine Kidney Cells with Human MDR1 Results in a Major Increase in Globotriaosylceramide and 105- to 106-Fold Increased Cell Sensitivity to Verocytotoxin. J. Biol. Chem. 275 (9), 6246–6251. 10.1074/jbc.275.9.6246 10692420

[B93] Lannert H. Gorgas K. Meissner I. Wieland F. T. Jeckel D. (1998). Functional Organization of the Golgi Apparatus in Glycosphingolipid Biosynthesis. J. Biol. Chem. 273 (5), 2939–2946. 10.1074/jbc.273.5.2939 9446606

[B94] Lau A. S. Hannigan G. E. Freedman M. H. Williams B. R. (1986). Regulation of Interferon Receptor Expression in Human Blood Lymphocytes *In Vitro* and during Interferon Therapy. J. Clin. Invest. 77 (5), 1632–1638. 10.1172/JCI112480 3009549 PMC424568

[B95] Lavie Y. Cao H.-t. Bursten S. L. Giuliano A. E. Cabot M. C. (1996). Accumulation of Glucosylceramides in Multidrug-Resistant Cancer Cells. J. Biol. Chem. 271 (32), 19530–19536. 10.1074/jbc.271.32.19530 8702646

[B96] Ledeen R. Wu G. (2011). New Findings on Nuclear Gangliosides: Overview on Metabolism and Function. J. Neurochem. 116 (5), 714–720. 10.1111/j.1471-4159.2010.07115.x 21214576

[B97] Lee H. C. Wondimu A. Liu Y. Ma J. S. Y. Radoja S. Ladisch S. (2012). Ganglioside Inhibition of CD8+T Cell Cytotoxicity: Interference with Lytic Granule Trafficking and Exocytosis. J.I. 189 (7), 3521–3527. 10.4049/jimmunol.1201256 22956583

[B98] Lee K.-H. Feig C. Tchikov V. Schickel R. Hallas C. Schütze S. (2006). The Role of Receptor Internalization in CD95 Signaling. EMBO J. 25 (5), 1009–1023. 10.1038/sj.emboj.7601016 16498403 PMC1409734

[B99] Lee K. S. Lee J. Lee P. Kim C. U. Kim D. J. Jeong Y. J. (2020). Exosomes Released from Shiga Toxin 2a-Treated Human Macrophages Modulate Inflammatory Responses and Induce Cell Death in Toxin Receptor Expressing Human Cells. Cell Microbiol. 22 (11), e13249. 10.1111/cmi.13249 32772454

[B100] Lee R.-S. Tartour E. van der Bruggen P. Vantomme V. Joyeux I. Goud B. (1998). Major Histocompatibility Complex Class I Presentation of Exogenous Soluble Tumor Antigen Fused to the B-Fragment of Shiga Toxin. Eur. J. Immunol. 28, 2726–2737. 10.1002/(SICI)1521-4141 9754560

[B101] Lee S.-Y. Lee M.-S. Cherla R. P. Tesh V. L. (2008). Shiga Toxin 1 Induces Apoptosis through the Endoplasmic Reticulum Stress Response in Human Monocytic Cells. Cell Microbiol 10 (3), 770–780. 10.1111/j.1462-5822.2007.01083.x 18005243

[B102] Legros N. Dusny S. Humpf H.-U. Pohlentz G. Karch H. Müthing J. (2017a). Shiga Toxin Glycosphingolipid Receptors and Their Lipid Membrane Ensemble in Primary Human Blood-Brain Barrier Endothelial Cells. Glycobiology 27 (1), 99–109. 10.1093/glycob/cww090 27558838

[B103] Legros N. Pohlentz G. Runde J. Dusny S. Humpf H.-U. Karch H. (2017b). Colocalization of Receptors for Shiga Toxins with Lipid Rafts in Primary Human Renal Glomerular Endothelial Cells and Influence of D-PDMP on Synthesis and Distribution of Glycosphingolipid Receptors. Glycobiology 27 (10), 947–965. 10.1093/glycob/cwx048 28535204

[B104] Levental I. Veatch S. L. (2016). The Continuing Mystery of Lipid Rafts. J. Mol. Biol. 428 (24 Pt A), 4749–4764. 10.1016/j.jmb.2016.08.022 27575334 PMC5124408

[B105] Li Y. Thapa P. Hawke D. Kondo Y. Furukawa K. Furukawa K. (2009). Immunologic Glycosphingolipidomics and NKT Cell Development in Mouse Thymus. J. Proteome Res. 8 (6), 2740–2751. 10.1021/pr801040h 19284783 PMC2720133

[B106] Liang Y.-J. Kuo H.-H. Lin C.-H. Chen Y.-Y. Yang B.-C. Cheng Y.-Y. (2010). Switching of the Core Structures of Glycosphingolipids from Globo- and Lacto- to Ganglio-Series upon Human Embryonic Stem Cell Differentiation. Proc. Natl. Acad. Sci. 107 (52), 22564–22569. 10.1073/pnas.1007290108 21149694 PMC3012484

[B107] Liang Y.-J. Yang B.-C. Chen J.-M. Lin Y.-H. Huang C.-L. Cheng Y.-Y. (2011). Changes in Glycosphingolipid Composition during Differentiation of Human Embryonic Stem Cells to Ectodermal or Endodermal Lineages. Stem Cells 29 (12), 1995–2004. 10.1002/stem.750 21956927

[B108] Lingwood C. A. Binnington B. Manis A. Branch D. R. (2010a). Globotriaosyl Ceramide Receptor Function - where Membrane Structure and Pathology Intersect. FEBS Lett. 584 (9), 1879–1886. 10.1016/j.febslet.2009.11.089 19948172

[B109] Lingwood C. A. Manis A. Mahfoud R. Khan F. Binnington B. Mylvaganam M. (2010b). New Aspects of the Regulation of Glycosphingolipid Receptor Function. Chem. Phys. Lipids 163 (1), 27–35. 10.1016/j.chemphyslip.2009.09.001 19781539

[B110] Lingwood C. A. Yiu S. K. (1992). Glycolipid Modification of α2 Interferon Binding. Sequence Similarity between the α2 Interferon Receptor and Verotoxin (Shiga-like Toxin) B-Subunit. Biochem. J. 283 (Pt 1), 25–26. 10.1042/bj2830025 1314564 PMC1130985

[B111] Lingwood C. (2020). Verotoxin Receptor-Based Pathology and Therapies. Front. Cel. Infect. Microbiol. 10, 123. 10.3389/fcimb.2020.00123 PMC7136409 32296648

[B112] Lingwood D. Simons K. (2010). Lipid Rafts as a Membrane-Organizing Principle. Science 327 (5961), 46–50. 10.1126/science.1174621 20044567

[B113] Liu Y.-Y. Gupta V. Patwardhan G. A. Bhinge K. Zhao Y. Bao J. (2010). Glucosylceramide Synthase Upregulates MDR1 Expression in the Regulation of Cancer Drug Resistance through cSrc and β-catenin Signaling. Mol. Cancer 9, 145. 10.1186/1476-4598-9-145 20540746 PMC2903501

[B114] Lombard J. López-García P. Moreira D. (2012). The Early Evolution of Lipid Membranes and the Three Domains of Life. Nat. Rev. Microbiol. 10 (7), 507–515. 10.1038/nrmicro2815 22683881

[B115] Lucci A. Cho W. I. Han T. Y. Giuliano A. E. Morton D. L. Cabot M. C. (1998). Glucosylceramide: a Marker for Multiple-Drug Resistant Cancers. Anticancer Res. 18 (1B), 475–480. 9568165

[B116] Ludwig A.-K. Vértesy S. Michalak M. C. Manning J. Andre S. Kubler D. (2016). Playing Modular Puzzle with Adhesion/Growth-Regulatory Galectins: Design and Testing of a Hybrid to Unravel Structure-Activity Relationships. Ppl 23 (11), 1003–1012. 10.2174/0929866523666160930123421 27697034

[B117] Madassery J. V. Gillard B. Marcus D. M. Nahm M. H. (1991). Subpopulations of B Cells in Germinal Centers. III. HJ6, a Monoclonal Antibody, Binds Globoside and a Subpopulation of Germinal center B Cells. J. Immunol. 147 (3), 823–829. 1713606

[B118] Maegawa G. H. B. van Giersbergen P. L. M. Yang S. Banwell B. Morgan C. P. Dingemanse J. (2009). Pharmacokinetics, Safety and Tolerability of Miglustat in the Treatment of Pediatric Patients with GM2 Gangliosidosis. Mol. Genet. Metab. 97 (4), 284–291. 10.1016/j.ymgme.2009.04.013 19447653

[B119] Mahfoud R. Manis A. Binnington B. Ackerley C. Lingwood C. A. (2010). A Major Fraction of Glycosphingolipids in Model and Cellular Cholesterol-Containing Membranes Is Undetectable by Their Binding Proteins. J. Biol. Chem. 285 (46), 36049–36059. 10.1074/jbc.M110.110189 20716521 PMC2975227

[B120] Majoul I. Schmidt T. Pomasanova M. Boutkevich E. Kozlov Y. Söling H. D. (2002). Differential Expression of Receptors for Shiga and Cholera Toxin Is Regulated by the Cell Cycle. J. Cel Sci 115 (Pt 4), 817–826. 10.1242/jcs.115.4.817 11865037

[B121] Maloney M. D. Lingwood C. A. (1994). CD19 Has a Potential CD77 (Globotriaosyl Ceramide)-Binding Site with Sequence Similarity to Verotoxin B-Subunits: Implications of Molecular Mimicry for B Cell Adhesion and Enterohemorrhagic *Escherichia coli* Pathogenesis. J. Exp. Med. 180 (1), 191–201. 10.1084/jem.180.1.191 7516406 PMC2191568

[B122] Mangeney M. Richard Y. Coulaud D. Tursz T. Wiels J. (1991). CD77: an Antigen of Germinal center B Cells Entering Apoptosis. Eur. J. Immunol. 21 (5), 1131–1140. 10.1002/eji.1830210507 1709864

[B123] Mannori G. Cecconi O. Mugnai G. Ruggieri S. (1990). Role of Glycolipids in the Metastatic Process: Characteristics of Neutral Glycolipids in Clones with Different Metastatic Potentials Isolated from a Murine Fibrosarcoma Cell Line. Int. J. Cancer 45 (5), 984–988. 10.1002/ijc.2910450535 2248638

[B124] Mathow D. Chessa F. Rabionet M. Kaden S. Jennemann R. Sandhoff R. (2015). Zeb1 Affects Epithelial Cell Adhesion by Diverting Glycosphingolipid Metabolism. EMBO Rep. 16 (3), 321–331. 10.15252/embr.201439333 25643708 PMC4364871

[B125] Matrosovich M. Herrler G. Klenk H. D. (2013). Sialic Acid Receptors of Viruses. Top. Curr. Chem. 367, 1–28. 10.1007/128_2013_466 PMC7120183 23873408

[B126] McEachern K. A. Fung J. Komarnitsky S. Siegel C. S. Chuang W.-L. Hutto E. (2007). A Specific and Potent Inhibitor of Glucosylceramide Synthase for Substrate Inhibition Therapy of Gaucher Disease. Mol. Genet. Metab. 91 (3), 259–267. 10.1016/j.ymgme.2007.04.001 17509920

[B127] McNab F. Mayer-Barber K. Sher A. Wack A. O'Garra A. (2015). Type I Interferons in Infectious Disease. Nat. Rev. Immunol. 15 (2), 87–103. 10.1038/nri3787 25614319 PMC7162685

[B128] Merrill A. H. Jr. (2011). Sphingolipid and Glycosphingolipid Metabolic Pathways in the Era of Sphingolipidomics. Chem. Rev. 111 (10), 6387–6422. 10.1021/cr2002917 21942574 PMC3191729

[B129] Miller J. J. Aoki K. Moehring F. Murphy C. A. O’Hara C. L. Tiemeyer M. (2018). Neuropathic Pain in a Fabry Disease Rat Model. JCI Insight 3 (6). 10.1172/jci.insight.99171 PMC5926911 29563343

[B130] Mizuguchi M. Tanaka S. Fujii I. Tanizawa H. Suzuki Y. Igarashi T. (1996). Neuronal and Vascular Pathology Produced by Verocytotoxin 2 in the Rabbit central Nervous System. Acta Neuropathologica 91 (3), 254–262. 10.1007/s004010050423 8834537

[B131] Mobassaleh M. Donohue-Rolfe A. Jacewicz M. Grand R. J. Keusch G. T. (1988). Pathogenesis of shigella Diarrhea: Evidence for a Developmentally Regulated Glycolipid Receptor for shigella Toxin Involved in the Fluid Secretory Response of Rabbit Small Intestine. J. Infect. Dis. 157 (5), 1023–1031. 10.1093/infdis/157.5.1023 3283253

[B132] Molostvov G. Morris A. Rose P. Basu S. (2001). Interaction of Cytokines and Growth Factor in the Regulation of Verotoxin-Induced Apoptosis in Cultured Human Endothelial Cells. Br. J. Haematol. 113 (4), 891–897. 10.1046/j.1365-2141.2001.02835.x 11442480

[B133] Moon D.-O. Choi S.-R. Lee C.-M. Kim G.-Y. Lee H.-J. Park Y.-M. (2005). Epigallocatechin-3-gallate Suppresses Galactose-Α1,4-Galactose-Β1,4-Glucose Ceramide Expression in TNF-α Stimulated Human Intestinal Epithelial Cells through Inhibition of MAPKs and NF-Κb. J. Korean Med. Sci. 20 (4), 548–554. 10.3346/jkms.2005.20.4.548 16100442 PMC2782146

[B134] Morace I. Pilz R. Federico G. Jennemann R. Krunic D. Nordström V. (2019). Renal Globotriaosylceramide Facilitates Tubular Albumin Absorption and its Inhibition Protects against Acute Kidney Injury. Kidney Int. 96 (2), 327–341. 10.1016/j.kint.2019.02.010 31101366

[B135] Morales A. Colell A. Mari M. Garcia-Ruiz C. Fernandez-Checa J. C. (2003). Glycosphingolipids and Mitochondria: Role in Apoptosis and Disease. Glycoconj J. 20 (9), 579–588. 10.1023/B:GLYC.0000043294.62504.2c 15454696

[B136] Moreau P. Bessoule J. J. Mongrand S. Testet E. Vincent P. Cassagne C. (1998). Lipid Trafficking in Plant Cells. Prog. Lipid Res. 37 (6), 371–391. 10.1016/s0163-7827(98)00016-2 10209654

[B137] Muanprasat C. Chatsudthipong V. (2013). Cholera: Pathophysiology and Emerging Therapeutic Targets. Future Med. Chem. 5 (7), 781–798. 10.4155/fmc.13.42 23651092

[B138] Naiki M. Kato M. (1979). Immunological Identification of Blood Group Pk Antigen on Normal Human Erythrocytes and Isolation of Anti-p^k with Different Affinity. Vox Sanguinis 37 (1), 30–38. 10.1111/j.1423-0410.1979.tb02265.x10.1159/000466879 494578

[B139] Nakayama H. Ogawa H. Takamori K. Iwabuchi K. (2013). GSL-enriched Membrane Microdomains in Innate Immune Responses. Arch. Immunol. Ther. Exp. 61 (3), 217–228. 10.1007/s00005-013-0221-6 23456206

[B140] Nicholson K. M. Quinn D. M. Kellett G. L. Warr J. R. (1999). Preferential Killing of Multidrug-Resistant KB Cells by Inhibitors of Glucosylceramide Synthase. Br. J. Cancer 81 (3), 423–430. 10.1038/sj.bjc.6690711 10507766 PMC2362922

[B141] Nishikawa K. Matsuoka K. Kita E. Okabe N. Mizuguchi M. Hino K. (2002). A Therapeutic Agent with Oriented Carbohydrates for Treatment of Infections by Shiga Toxin-Producing *Escherichia coli* O157:H7. Proc. Natl. Acad. Sci. 99 (11), 7669–7674. 10.1073/pnas.112058999 12032341 PMC124317

[B142] Obata F. (2010). Influence of *Escherichia coli* Shiga Toxin on the Mammalian central Nervous System. Adv. Appl. Microbiol. 71, 1–19. 10.1016/S0065-2164(10)71001-7 20378049

[B143] Obata F. Obrig T. (2010). Distribution of Gb3 Immunoreactivity in the Mouse Central Nervous System. Toxins 2 (8), 1997–2006. 10.3390/toxins2081997 20725533 PMC2923496

[B144] Obata F. Tohyama K. Bonev A. D. Kolling G. L. Keepers T. R. Gross L. K. (2008). Shiga Toxin 2 Affects the central Nervous System through Receptor Globotriaosylceramide Localized to Neurons. J. Infect. Dis. 198 (9), 1398–1406. 10.1086/591911 18754742 PMC2684825

[B145] Okuda T. Nakayama K.-i. (2008). Identification and Characterization of the Human Gb3/CD77 Synthase Gene Promoter. Glycobiology 18 (12), 1028–1035. 10.1093/glycob/cwn082 18757779

[B146] Okuda T. Tokuda N. Numata S.-i. Ito M. Ohta M. Kawamura K. (2006). Targeted Disruption of Gb3/CD77 Synthase Gene Resulted in the Complete Deletion of Globo-Series Glycosphingolipids and Loss of Sensitivity to Verotoxins. J. Biol. Chem. 281 (15), 10230–10235. 10.1074/jbc.M600057200 16476743

[B147] Pacheco A. R. Lazarus J. E. Sit B. Schmieder S. Lencer W. I. Blondel C. J. (2018). CRISPR Screen Reveals that EHEC's T3SS and Shiga Toxin Rely on Shared Host Factors for Infection. mBio 9 (3). 10.1128/mBio.01003-18 PMC6016243 29921669

[B148] Palm W. (2021). GOLPH3 Tunes up Glycosphingolipid Biosynthesis for Cell Growth. EMBO J. 40 (8), e108070. 10.15252/embj.2021108070 33763859 PMC8047436

[B149] Papanikou E. Glick B. S. (2014). Golgi Compartmentation and Identity. Curr. Opin. Cel Biol. 29, 74–81. 10.1016/j.ceb.2014.04.010 PMC4130901 24840895

[B150] Pascual V. Liu Y. J. Magalski A. de Bouteiller O. Banchereau J. Capra J. D. (1994). Analysis of Somatic Mutation in Five B Cell Subsets of Human Tonsil. J. Exp. Med. 180 (1), 329–339. 10.1084/jem.180.1.329 8006591 PMC2191579

[B151] Percec V. Leowanawat P. Sun H.-J. Kulikov O. Nusbaum C. D. Tran T. M. (2013). Modular Synthesis of Amphiphilic Janus Glycodendrimers and Their Self-Assembly into Glycodendrimersomes and Other Complex Architectures with Bioactivity to Biomedically Relevant Lectins. J. Am. Chem. Soc. 135 (24), 9055–9077. 10.1021/ja403323y 23692629

[B152] Pestka S. Krause C. D. Walter M. R. (2004). Interferons, Interferon-like Cytokines, and Their Receptors. Immunol. Rev. 202, 8–32. 10.1111/j.0105-2896.2004.00204.x 15546383

[B153] Peterschmitt M. J. Burke A. Blankstein L. Smith S. E. Puga A. C. Kramer W. G. (2011). Safety, Tolerability, and Pharmacokinetics of Eliglustat Tartrate (Genz-112638) after Single Doses, Multiple Doses, and Food in Healthy Volunteers. J. Clin. Pharmacol. 51 (5), 695–705. 10.1177/0091270010372387 20864621

[B154] Petit D. Teppa R. E. Harduin-Lepers A. (2021). A Phylogenetic View and Functional Annotation of the Animal β1,3-glycosyltransferases of the GT31 CAZy Family. Glycobiology 31 (3), 243–259. 10.1093/glycob/cwaa086 32886776 PMC8022947

[B155] Petroutsos D. Amiar S. Abida H. Dolch L.-J. Bastien O. Rébeillé F. (2014). Evolution of Galactoglycerolipid Biosynthetic Pathways - from Cyanobacteria to Primary Plastids and from Primary to Secondary Plastids. Prog. Lipid Res. 54, 68–85. 10.1016/j.plipres.2014.02.001 24594266

[B156] Pinto A. Cangelosi A. Geoghegan P. A. Goldstein J. (2017). Dexamethasone Prevents Motor Deficits and Neurovascular Damage Produced by Shiga Toxin 2 and Lipopolysaccharide in the Mouse Striatum. Neuroscience 344, 25–38. 10.1016/j.neuroscience.2016.12.036 28042026

[B157] Pinto A. Jacobsen M. Geoghegan P. A. Cangelosi A. Cejudo M. L. Tironi-Farinati C. (2013). Dexamethasone Rescues Neurovascular Unit Integrity from Cell Damage Caused by Systemic Administration of Shiga Toxin 2 and Lipopolysaccharide in Mice Motor Cortex. PLoS One 8 (7), e70020. 10.1371/journal.pone.0070020 23894578 PMC3720947

[B158] Pothukuchi P. Agliarulo I. Pirozzi M. Rizzo R. Russo D. Turacchio G. (2021). GRASP55 Regulates intra‐Golgi Localization of Glycosylation Enzymes to Control Glycosphingolipid Biosynthesis. EMBO J. 40 (20), e107766. 10.15252/embj.2021107766 34516001 PMC8521277

[B159] Prinetti A. Loberto N. Chigorno V. Sonnino S. (2009). Glycosphingolipid Behaviour in Complex Membranes. Biochim. Biophys. Acta (Bba) - Biomembranes 1788 (1), 184–193. 10.1016/j.bbamem.2008.09.001 18835549

[B160] Provençal P. Boutin M. Dworski S. Au B. Medin J. A. Auray-Blais C. (2016). Relative Distribution of Gb3 Isoforms/analogs in NOD/SCID/Fabry Mice Tissues Determined by Tandem Mass Spectrometry. Bioanalysis 8 (17), 1793–1807. 10.4155/bio-2016-0116 27523577 PMC4992964

[B161] Psotka M. A. Obata F. Kolling G. L. Gross L. K. Saleem M. A. Satchell S. C. (2009). Shiga Toxin 2 Targets the Murine Renal Collecting Duct Epithelium. Infect. Immun. 77 (3), 959–969. 10.1128/IAI.00679-08 19124603 PMC2643625

[B162] Pudymaitis A. Lingwood C. A. (1992). Susceptibility to Verotoxin as a Function of the Cell Cycle. J. Cel. Physiol. 150 (3), 632–639. 10.1002/jcp.1041500324 1537890

[B163] Ramegowda B. Tesh V. L. (1996). Differentiation-associated Toxin Receptor Modulation, Cytokine Production, and Sensitivity to Shiga-like Toxins in Human Monocytes and Monocytic Cell Lines. Infect. Immun. 64 (4), 1173–1180. 10.1128/iai.64.4.1173-1180.1996 8606075 PMC173900

[B164] Regina Todeschini A. Hakomori S.-i. (2008). Functional Role of Glycosphingolipids and Gangliosides in Control of Cell Adhesion, Motility, and Growth, through Glycosynaptic Microdomains. Biochim. Biophys. Acta (Bba) - Gen. Subjects 1780 (3), 421–433. 10.1016/j.bbagen.2007.10.008 PMC2312458 17991443

[B165] Ren J. Utsunomiya I. Taguchi K. Ariga T. Tai T. Ihara Y. (1999). Localization of Verotoxin Receptors in Nervous System. Brain Res. 825 (1-2), 183–188. 10.1016/s0006-8993(99)01196-8 10216186

[B166] Richardson S. E. Rotman T. A. Jay V. Smith C. R. Becker L. E. Petric M. (1992). Experimental Verocytotoxemia in Rabbits. Infect. Immun. 60 (10), 4154–4167. 10.1128/iai.60.10.4154-4167.1992 1398926 PMC257448

[B167] Ridley A. (2000). Molecular Switches in Metastasis. Nature 406 (6795), 466–467. 10.1038/35020170 10952292

[B168] Rizzo R. Russo D. Kurokawa K. Sahu P. Lombardi B. Supino D. (2021). Golgi Maturation‐dependent Glycoenzyme Recycling Controls Glycosphingolipid Biosynthesis and Cell Growth via GOLPH3. EMBO J. 40 (8), e107238. 10.15252/embj.2020107238 33749896 PMC8047446

[B169] Roy K. R. Uddin M. B. Roy S. C. Hill R. A. Marshall J. Li Y. T. (2020). Gb3‐cSrc Complex in Glycosphingolipid‐enriched Microdomains Contributes to the Expression of P53 Mutant Protein and Cancer Drug Resistance via β‐catenin-activated RNA Methylation. FASEB BioAdvances 2 (11), 653–667. 10.1096/fba.2020-00044 33205006 PMC7655095

[B170] Ruiz F. M. Scholz B. A. Buzamet E. Kopitz J. André S. Menéndez M. (2014). Natural Single Amino Acid Polymorphism (F19Y) in Human Galectin‐8: Detection of Structural Alterations and Increased Growth‐regulatory Activity on Tumor Cells. FEBS J. 281 (5), 1446–1464. 10.1111/febs.12716 24418318

[B171] Russo D. Della Ragione F. Rizzo R. Sugiyama E. Scalabrì F. Hori K. (2018). Glycosphingolipid Metabolic Reprogramming Drives Neural Differentiation. EMBO J. 37 (7). 10.15252/embj.201797674 PMC5881633 29282205

[B172] Salhia B. Rutka J. T. Lingwood C. Nutikka A. Van Furth W. R. (2002). The Treatment of Malignant Meningioma with Verotoxin. Neoplasia 4 (4), 304–311. 10.1038/sj.neo.7900243 12082546 PMC1531702

[B173] Sánchez D. S. Fischer Sigel L. K. Balestracci A. Ibarra C. Amaral M. M. Silberstein C. (2021). Eliglustat Prevents Shiga Toxin 2 Cytotoxic Effects in Human Renal Tubular Epithelial Cells. Pediatr. Res. 10.1038/s41390-021-01622-3 34155339

[B174] Sato W. Watanabe-Takahashi M. Hamabata T. Furukawa K. Funamoto S. Nishikawa K. (2021). A Nontoxigenic Form of Shiga Toxin 2 Suppresses the Production of Amyloid β by Altering the Intracellular Transport of Amyloid Precursor Protein through its Receptor-Binding B-Subunit. Biochem. Biophysical Res. Commun. 557, 247–253. 10.1016/j.bbrc.2021.04.015 33894410

[B175] Schnaar R. L. Suzuki A. Stanley P. (2009). “Glycosphingolipids,” in Essentials of Glycobiology. 2nd ed (New York: Press).

[B176] Schüller S. (2011). Shiga Toxin Interaction with Human Intestinal Epithelium. Toxins 3 (6), 626–639. 10.3390/toxins3060626 22069729 PMC3202847

[B177] Schwatz-Albiez R. Dörken B. Möller P. Brodin N. T. Monner D. A. Kniep B. (1990). Neutral Glycosphingolipids of the Globo-Series Characterize Activation Stages Corresponding to Germinal center B Cells. Int. Immunol. 2 (10), 929–936. 10.1093/intimm/2.10.929 2078521

[B178] Shayman J. A. Lee L. Abe A. Shu L. (2000). [38] Inhibitors of Glucosylceramide Synthase. Methods Enzymol. 311, 373–387. 10.1016/s0076-6879(00)11097-3 10563341

[B179] Shimizu H. (2003). Studies on Molecular Interactions of Protein and Their Carbohydrate Ligands by NMR. Trends Glycoscience Glycotechnology 15 (84), 221–233. 10.4052/tigg.15.221

[B180] Shin I.-S. Ishii S. Shin J.-S. Sung K.-I. Park B.-S. Jang H.-Y. (2009). Globotriaosylceramide (Gb3) Content in HeLa Cells Is Correlated to Shiga Toxin-Induced Cytotoxicity and Gb3 Synthase Expression. BMB Rep. 42 (5), 310–314. 10.5483/bmbrep.2009.42.5.310 19470247

[B181] Silberstein C. Lucero M. S. Zotta E. Copeland D. P. Lingyun L. Repetto H. A. (2011). A Glucosylceramide Synthase Inhibitor Protects Rats against the Cytotoxic Effects of Shiga Toxin 2. Pediatr. Res. 69 (5 Pt 1), 390–394. 10.1203/PDR.0b013e318211dd57 21270676

[B182] Simons K. Toomre D. (2000). Lipid Rafts and Signal Transduction. Nat. Rev. Mol. Cel Biol 1 (1), 31–39. 10.1038/35036052 11413487

[B183] Solís D. Bovin N. V. Davis A. P. Jiménez-Barbero J. Romero A. Roy R. (2015). A Guide into Glycosciences: How Chemistry, Biochemistry and Biology Cooperate to Crack the Sugar Code. Biochim. Biophys. Acta (Bba) - Gen. Subjects 1850 (1), 186–235. 10.1016/j.bbagen.2014.03.016 24685397

[B184] Ståhl A.-l. Arvidsson I. Johansson K. E. Chromek M. Rebetz J. Loos S. (2015). A Novel Mechanism of Bacterial Toxin Transfer within Host Blood Cell-Derived Microvesicles. Plos Pathog. 11 (2), e1004619. 10.1371/journal.ppat.1004619 25719452 PMC4342247

[B185] Stein K. E. Marcus D. M. (1977). Glycosphingolipids of Purified Human Lymphocytes. Biochemistry 16 (24), 5285–5291. 10.1021/bi00643a019 303523

[B186] Stimmer L. Dehay S. Nemati F. Massonnet G. Richon S. Decaudin D. (2014). Human Breast Cancer and Lymph Node Metastases Express Gb3 and Can Be Targeted by STxB-Vectorized Chemotherapeutic Compounds. BMC Cancer 14, 916. 10.1186/1471-2407-14-916 25476116 PMC4289340

[B187] Suzuki K. G. N. (2015). New Insights into the Organization of Plasma Membrane and its Role in Signal Transduction. Int. Rev. Cel Mol Biol 317, 67–96. 10.1016/bs.ircmb.2015.02.004 26008784

[B188] Takahashi K. Funata N. Ikuta F. Sato S. (2008). Neuronal Apoptosis and Inflammatory Responses in the central Nervous System of a Rabbit Treated with Shiga Toxin-2. J. Neuroinflammation 5, 11. 10.1186/1742-2094-5-11 18355415 PMC2330034

[B189] Tan A. H.-M. Sanny A. Ng S.-W. Ho Y.-S. Basri N. Lee A. P. (2018). Excessive Interferon-α Signaling in Autoimmunity Alters Glycosphingolipid Processing in B Cells. J. Autoimmun. 89, 53–62. 10.1016/j.jaut.2017.11.004 29191573

[B190] Tedder T. F. Zhou L.-J. Engel P. (1994). The CD19/CD21 Signal Transduction Complex of B Lymphocytes. Immunol. Today 15 (9), 437–442. 10.1016/0167-5699(94)90274-7 7524521

[B191] Tironi-Farinati C. Loidl C. F. Boccoli J. Parma Y. Fernandez-Miyakawa M. E. Goldstein J. (2010). Intracerebroventricular Shiga Toxin 2 Increases the Expression of its Receptor Globotriaosylceramide and Causes Dendritic Abnormalities. J. Neuroimmunology 222 (1-2), 48–61. 10.1016/j.jneuroim.2010.03.001 20347160

[B192] Trachtman H. Cnaan A. Christen E. Gibbs K. Zhao S. Acheson D. W. (2003). Effect of an Oral Shiga Toxin-Binding Agent on Diarrhea-Associated Hemolytic Uremic Syndrome in ChildrenA Randomized Controlled Trial. JAMA 290 (10), 1337–1344. 10.1001/jama.290.10.1337 12966125

[B193] Tyler A. Johansson A. Karlsson T. Gudey S. K. Brännström T. Grankvist K. (2015). Targeting Glucosylceramide Synthase Induction of Cell Surface Globotriaosylceramide (Gb3) in Acquired Cisplatin-Resistance of Lung Cancer and Malignant Pleural Mesothelioma Cells. Exp. Cel Res. 336 (1), 23–32. 10.1016/j.yexcr.2015.05.012 26004871

[B194] Uchida H. Kiyokawa N. Taguchi T. Horie H. Fujimoto J. Takeda T. (1999). Shiga Toxins Induce Apoptosis in Pulmonary Epithelium-Derived Cells. J. Infect. Dis. 180 (6), 1902–1911. 10.1086/315131 10558947

[B195] Utsunomiya I. Ren J. Taguchi K. Ariga T. Tai T. Ihara Y. (2001). Immunohistochemical Detection of Verotoxin Receptors in Nervous System. Brain Res. Protoc. 8 (2), 99–103. 10.1016/s1385-299x(01)00091-5 11673091

[B196] Uzzo R. G. Rayman P. Kolenko V. Clark P. E. Cathcart M. K. Bloom T. (1999). Renal Cell Carcinoma-Derived Gangliosides Suppress Nuclear Factor-Κb Activation in T Cells. J. Clin. Invest. 104 (6), 769–776. 10.1172/JCI6775 10491412 PMC408430

[B197] Varki A. Freeze H. H. Gagneux P. (2009). in Evolution of Glycan DiversityEssentials of Glycobiology. 2nd ed (New York: Press).

[B198] Vengris V. E. Reynolds F. H. Jr. Hollenberg M. D. Pitha P. M. (1976). Interferon Action: Role of Membrane Gangliosides. Virology 72 (2), 486–493. 10.1016/0042-6822(76)90177-x 181911

[B199] Wegner M.-S. Gruber L. Mattjus P. Geisslinger G. Grösch S. (2018a). The UDP-Glucose Ceramide Glycosyltransferase (UGCG) and the Link to Multidrug Resistance Protein 1 (MDR1). BMC Cancer 18 (1), 153. 10.1186/s12885-018-4084-4 29409484 PMC5801679

[B200] Wegner M.-S. Schömel N. Gruber L. Örtel S. B. Kjellberg M. A. Mattjus P. (2018b). UDP-glucose Ceramide Glucosyltransferase Activates AKT, Promoted Proliferation, and Doxorubicin Resistance in Breast Cancer Cells. Cell. Mol. Life Sci. 75 (18), 3393–3410. 10.1007/s00018-018-2799-7 29549423 PMC11105721

[B201] Wiels J. Holmes E. H. Cochran N. Tursz T. Hakomori S. (1984). Enzymatic and Organizational Difference in Expression of a Burkitt Lymphoma-Associated Antigen (Globotriaosylceramide) in Burkitt Lymphoma and Lymphoblastoid Cell Lines. J. Biol. Chem. 259 (23), 14783–14787. 10.1016/s0021-9258(17)42671-8 6438103

[B202] Willysson A. Ståhl A.-l. Gillet D. Barbier J. Cintrat J.-C. Chambon V. (2020). Shiga Toxin Uptake and Sequestration in Extracellular Vesicles Is Mediated by its B-Subunit. Toxins 12 (7), 449. 10.3390/toxins12070449 PMC7404996 32664382

[B203] Xiao Q. Zhang S. Wang Z. Sherman S. E. Moussodia R.-O. Peterca M. (2016). Onion-like Glycodendrimersomes from Sequence-Defined Janus Glycodendrimers and Influence of Architecture on Reactivity to a Lectin. Proc. Natl. Acad. Sci. USA 113 (5), 1162–1167. 10.1073/pnas.1524976113 26787853 PMC4747702

[B204] Xu H. Peng L. Shen M. Xia Y. Li Z. He N. (2019). Shiga‐like Toxin I Exerts Specific and Potent Anti‐tumour Efficacy against Gastric Cancer Cell Proliferation when Driven by Tumour‐preferential Frizzled‐7 Promoter. Cell Prolif 52 (3), e12607. 10.1111/cpr.12607 30955216 PMC6536451

[B205] Yamada A. Arakaki R. Saito M. Kudo Y. Ishimaru N. (2017). Dual Role of Fas/FasL-Mediated Signal in Peripheral Immune Tolerance. Front. Immunol. 8, 403. 10.3389/fimmu.2017.00403 28424702 PMC5380675

[B206] Yan N. Chen Z. J. (2012). Intrinsic Antiviral Immunity. Nat. Immunol. 13 (3), 214–222. 10.1038/ni.2229 22344284 PMC3549670

[B207] Yang Z. Bergström J. Karlsson K. A. (1994). Glycoproteins with Gal Alpha 4Gal Are Absent from Human Erythrocyte Membranes, Indicating that Glycolipids Are the Sole Carriers of Blood Group P Activities. J. Biol. Chem. 269 (20), 14620–14624. 10.1016/s0021-9258(17)36669-3 8182069

[B208] Yoon H. J. Jeon S. B. Suk K. Choi D. K. Hong Y. J. Park E. J. (2008). Contribution of TLR2 to the Initiation of Ganglioside-Triggered Inflammatory Signaling. Mol. Cell 25 (1), 99–104. 18319620

[B209] Zhang S. Moussodia R.-O. Murzeau C. Sun H.-J. Klein M. L. Vértesy S. (2015a). Dissecting Molecular Aspects of Cell Interactions Using Glycodendrimersomes with Programmable Glycan Presentation and Engineered Human Lectins. Angew. Chem. Int. Ed. 54 (13), 4036–4040. 10.1002/anie.201410882 25656452

[B210] Zhang S. Moussodia R.-O. Vértesy S. André S. Klein M. L. Gabius H.-J. (2015b). Unraveling Functional Significance of Natural Variations of a Human Galectin by Glycodendrimersomes with Programmable Glycan Surface. Proc. Natl. Acad. Sci. USA 112 (18), 5585–5590. 10.1073/pnas.1506220112 25902539 PMC4426414

[B211] Zhang T. de Waard A. A. Wuhrer M. Spaapen R. M. (2019). The Role of Glycosphingolipids in Immune Cell Functions. Front. Immunol. 10, 90. 10.3389/fimmu.2019.00090 30761148 PMC6361815

[B212] Zhang X. Kiechle F. L. (2004). Review: Glycosphingolipids in Health and Disease. Ann. Clin. Lab. Sci. 34 (1), 3–13. 15038664

